# A Micro‐Flow Liquid Chromatography–Mass Spectrometry Method for the Quantification of Oxylipins in Volume‐Limited Human Plasma

**DOI:** 10.1002/elps.202400151

**Published:** 2024-11-11

**Authors:** Bingshu He, Rawi Ramautar, Marian Beekman, P. Eline Slagboom, Amy Harms, Thomas Hankemeier

**Affiliations:** ^1^ Metabolomics and Analytics Centre Leiden Academic Centre for Drug Research (LACDR) Leiden University Leiden The Netherlands; ^2^ Department of Molecular Epidemiology Leiden University Medical Centre Leiden The Netherlands

**Keywords:** micro–liquid chromatography (LC)–mass spectrometry (MS), oxylipins, sensitivity enhancement, volume‐limited plasma

## Abstract

Oxylipins are well‐known lipid mediators in various inflammatory conditions. Their endogenous concentrations range from low picomolar to nanomolar, and there are growing demands to determine their concentrations in low‐volume matrices for pathological studies, including blood, cerebrospinal fluids from animal disease models, infants, and microsampling devices. Most of the published quantification methods for comprehensive profiling of oxylipins still require more than 50 µL plasma as a starting volume to detect these low levels. The aim of our study is to develop a sensitive and reliable method for the quantification of oxylipins in volume‐limited human plasma samples. We established and validated a micro–liquid chromatography (LC)–mass spectrometry (MS)/MS method that requires only 5 µL of human plasma for the determination of 66 oxylipins. The optimized micro‐LC–MS/MS method utilized a flow rate of 4 µL/min with a 0.3‐mm inner diameter column. With an injection volume of 3 µL, our method provides limits of detection in the range from 0.1 to 91.9 pM, and limits of quantification range from 0.3 to 306.2 pM. The sensitivity enhancement compared to conventional flow ranged from 1.4 to 180.7 times for 51 compounds depending on their physical–chemical properties. After validation, the method was applied to analyze 40 plasma samples from a healthy aging study to demonstrate robustness and sensitivity.

**Abbreviations**
AA: arachidonic acid
BHT: butylated hydroxytoluene
BuOH: 1‐butanol
EMA: European Medicines Agency
EPA: eicosapentaenoic acid
ISTD: internal standard
LLE: liquid–liquid extraction
MeOH: methanol
MTBE: methyl *tert*‐butyl ether
PGs: prostaglandins
PUFAs: polyunsaturated fatty acids
QC: quality control
SRM: selected reaction monitoring

## 1 Introduction

Oxylipins are a group of signaling lipids derived from polyunsaturated fatty acids (PUFAs) through enzymatic or non‐enzymatic oxidation processes. Non‐esterified or free oxylipins function as important lipid mediators in multiple physiological processes as well as disease pathologies. Cardiovascular diseases, obesity, type II diabetes, and neurological disorders have been linked to abnormal oxylipin signaling [1, 2]. Oxylipins include some well‐studied subclasses, such as prostaglandins (PGs), arachidonic acid (AA)‐derived isoprostanes, AA‐ and eicosapentaenoic acid (EPA)‐derived prostanoids, AA‐derived leukotrienes, and lipoxins, which are involved in inflammation, blood clotting, immune response, cardiovascular diseases, and tumor progression [3, 4, 5, 6, 7].

As the most direct and easy to sample matrix, plasma is commonly utilized for studying the onset and progression of pathological processes. Despite the increasing demand for oxylipin profiling and understanding their functions, currently available liquid chromatography–mass spectrometry (LC–MS) methods for the quantification of PUFAs and their derivatives often fall short when dealing with volume‐limited plasma samples [8, 9, 10, 11]. Although plasma from adults is not normally recognized as volume‐limited, when one sample set has to be aliquoted to multiple analytical platforms for a comprehensive multi‐omics analysis, only a limited volume is available for each platform. On the other hand, the sampling techniques in healthcare favor sampling low volumes (below 10 µL) to ease the pain for patients [12, 13] and improve the wellness of experimental animals [14]. The issue of volume mismatch between sampling and sample analysis can be addressed by employing a miniaturized LC–MS workflow, which allows high detection sensitivity with a minimal sample volume.

One example of how a miniaturized workflow can increase sensitivity was presented by Cebo et al. [15] who established a micro‐LC–MS/MS method for accurate quantification of 42 oxylipins in plasma using a flow rate of 30 µL/min. Although the method allowed for the quantitation of some oxylipins down to the picogram level, a relatively large volume of plasma (500 µL) was used, and the sample was up‐concentrated to 100 µL to achieve the required detection limits. A further down‐scaling in flow rate may lead to higher sensitivity, which enables the quantification of oxylipins using less starting material.

In this study, we established a micro‐LC–MS workflow for the quantification of 66 oxylipins in 5 µL human plasma. Selected reaction monitoring (SRM) was applied in a targeted method in negative ion mode. Multiple electrospray ionization sources were compared, corona discharge was minimized, and analytical performance was optimized. A reverse phase C18 column with a 0.3‐mm inner diameter was used for this method at a flow rate of 4 µL/min. The method underwent validation following the bioanalytical method validation guidelines from EMA (2009). The sensitivity enhancement was evaluated by comparing the limits of detection (LODs) and limits of quantification (LOQs) with a published conventional flow UHPLC–MS method [16]. The validated method was applied to 40 human plasma samples from a healthy aging study with 5 µL as the starting volume. A total of 28 oxylipins passed our quality criteria and were quantified from picomolar to nanomolar level. This underscores its potential value for future studies that demand high sensitivity in determining oxylipin concentrations in volume‐limited samples.

## 2 Materials and Methods

### 2.1 Chemicals and Materials

Ultra‐performance liquid chromatography grade acetonitrile (ACN) and methanol (MeOH) were purchased from Biosolve (Valkenswaard, The Netherlands). 1‐Butanol (BuOH) was acquired from Boom (Meppel, The Netherlands). Methyl *tert*‐butyl ether (MTBE), acetic acid (100%, LC–MS grade), butylated hydroxytoluene (BHT), and ethylenediaminetetraacetic acid (EDTA) were from Sigma Aldrich (Zwijndrecht, The Netherlands). Citric acid monohydrate and disodium hydrogen phosphate were obtained from Merck (Darmstadt, Germany). Purified water was obtained from a Milli‐*Q* PF Plus system (Merck Millipore, Burlington, Massachusetts, USA). Standards and deuterated standards for oxylipins were purchased from Cayman Chemicals (Ann Arbor, Michigan, USA). The purchasing information and abbreviations of all the targeted oxylipins and labeled standards are listed in Table S1.

### 2.2 Preparation of Standards Solutions and Calibrant Solutions

Stock solutions of oxylipins and labeled standards were dissolved in MeOH containing BHT (0.4 mg/mL) to 1 mg/mL. The oxylipin standard stock solutions were diluted to 11 calibrant points (C1–C11) subsequently. Internal standards (ISTDs) were spiked in each calibrant point and sample to reach a final concentration of 20 nM. All the stock solutions were stored at −20°C. The compound list with their calibration concentrations can be found in Table S2.

### 2.3 Sample Collection and Preparation

Fasted EDTA plasma samples were collected from 40 participants in the Leiden Longevity Study cohort, which was enrolled between 2002 and 2006 [17]. The samples for the current study were collected at the third visit between 2009 and 2010 and stored at −80°C. The Leiden Longevity Study protocol (P09.140) was approved by the Medical Ethical Committee of the Leiden University Medical Center before the start of the study at August 5, 2009. The first participant was enrolled at August 24, 2009. In accordance with the Declaration of Helsinki, the Leiden Longevity Study obtained informed consent from all participants prior to their entering the study. Five microliters was taken from each plasma sample and pooled together to create a quality control (QC) sample used during method validation and bioanalysis. All plasma samples remained at −80°C until extraction.

Plasma samples, calibration samples, and QCs were thawed on ice before liquid–liquid extraction (LLE). Plasma of 5 µL (or calibrant solution), antioxidant solution of 5 µL, 5 µL 80 nM ISTD, buffer solution of 5 µL containing 0.2 M citric acid, and 0.4 M phosphate at pH 4.5 were added in a 0.5 mL Eppendorf tube. After adding 100 µL BuOH:MTBE (1:1, v/v) and 50 µL water, oxylipins were extracted to the upper layer. Subsequently, the samples were settled on ice for 20 min. This will facilitate the LLE because the protein layer will become more compact. After that, the samples were brought to a bullet blender to be thoroughly mixed for 4 min at speed level 9. Samples were then centrifuged for 10 min at 15 800 rcf under 4°C. The organic upper layer of 85 µL was collected and evaporated to dryness. Samples were reconstituted in a 20 µL mixture of MeOH:H_2_O (2:8, v/v) before injection.

### 2.4 Micro‐LC–MS/MS Instrumentation and Conditions

The micro‐LC–MS/MS analyses were performed using a Waters nanoAcquity LC system (Waters, Milford, Massachusetts, USA) coupled to a Sciex triple quad 7500 mass spectrometer (SCIEX, Framingham, Massachusetts, US). A Sciex OptiFlow ionization source was equipped on the MS with a SteadySpray Low Micro Electrode (1–50 µL/min). The separation was carried out using a Phenomenex Kinetex C18 column (2.6 µm, 150 × 0.3 mm^2^) maintained at 40°C. The injection volume was 3 µL. Mobile phase A consisted of 0.1% acetic acid in water; mobile phase B was 0.1% acetic acid in a mixture of 90% ACN and 10% MeOH. Using a flow rate of 4 µL/min, the initial gradient started at 20% eluent‐B and maintained for 5.0 min, eluent‐B was ramped to 26% until 5.4 min, and further to 34% at 15.5 min, remained at 34% for 2 min and increased to 40% at 19.5 min, to 54% at 23.5 min, to 56% at 27.5 min, to 78% at 29.5 min, to 85% at 31.5 min, at last eluent‐B dropped back to 20% for re‐equilibration until 40.0 min. MS data were acquired in negative ionization mode with curtain gas flow rate 32 psi, gas 1 flow rate 10 psi, gas 2 flow rate 20 psi, interface voltage at −3500 V, and interface temperature at 200°C. SRM was used for data acquisition by monitoring the precursor–product ion transitions as indicated in Table S3. These instrumental conditions were used during method validation and application to plasma samples.

### 2.5 Method Validation

#### 2.5.1 Linearity and Limit of Detection

To assess linearity, calibration lines (*n* = 3) were prepared over 3 consecutive days, incorporating a minimum of seven calibration points for each compound. All calibration lines were fitted to a 1/*x* ^2^ weighted linear regression model. The LODs and LOQs were calculated using the formulas LOD = 3 × *S*_a_/*b* and LOQ = 10 × *S*_a_/*b*, respectively. Here, *S*_a_ represents the standard deviation of the *y*‐intercept, and *b* represents the slope of the calibration curve.

#### 2.5.2 Precision

The intra‐ and interday precisions were evaluated by spiking three different concentrations of ISTD solutions (low‐level [0.2 nM], medium‐level [2.0 nM], and high‐level [20 nM]) into pooled plasma samples over 3 different days (*n* = 3). Precision was expressed as the RSD of the peak areas of ISTD. An RSD less than 15% was within the tolerance limits of the EMA guidelines [18].

#### 2.5.3 Recovery and Matrix Effects

Recovery was investigated by spiking ISTD solution before LLE and after LLE. Matrix effect was evaluated by spiking ISTD solutions to pooled plasma samples (*n* = 3) or water (*n* = 3). Recovery was calculated as the ratio of ISTD peak areas spiked before and after extraction. The matrix effect was determined by calculating the ratio of the peak areas of ISTD spiked in pooled plasma and water samples.

### 2.6 Comparison to Conventional UHPLC–MS/MS Method

The performance of the optimized micro‐LC–MS/MS method was compared to a conventional UHPLC–MS/MS described in a previous study [16]. The sample preparation procedure and starting volume (5 µL plasma) were the same in both methods as described in Section 2.3. A Shimadzu Nexera X2 LC‐30AD system (Shimadzu Corporation, Kyoto, Japan) was coupled to a SCIEX triple quad 7500 mass spectrometer (SCIEX, Framingham, Massachusetts, USA). An OptiFlow ionization source with spray emitters for high flow (>200 µL/min) was employed. The separation was carried out on a Waters Acquity BEH C18 column (50 × 2.1 mm^2^, 1.7 µm) maintained at 40°C with a 5‐µL injection volume. The detailed UHPLC gradient can be found in Table S4. MS data were acquired in negative ionization mode with a curtain gas flow rate of 45 psi, gas 1 flow rate of 65 psi, gas 2 flow rate of 65 psi, an interface voltage of −4500 V, and an interface temperature of 600°C.

### 2.7 Data Preprocessing

SCIEX OS version 1.6 (SCIEX, USA) was used for peak integration. Absolute quantitation was calculated using the equation of the calibration curve and using the peak area ratios (peak area of targeted analyte divided by peak area of respective ISTDs).

## 3 Results and Discussion

### 3.1 Micro‐LC–MS/MS Method Development

**FIGURE 1:**
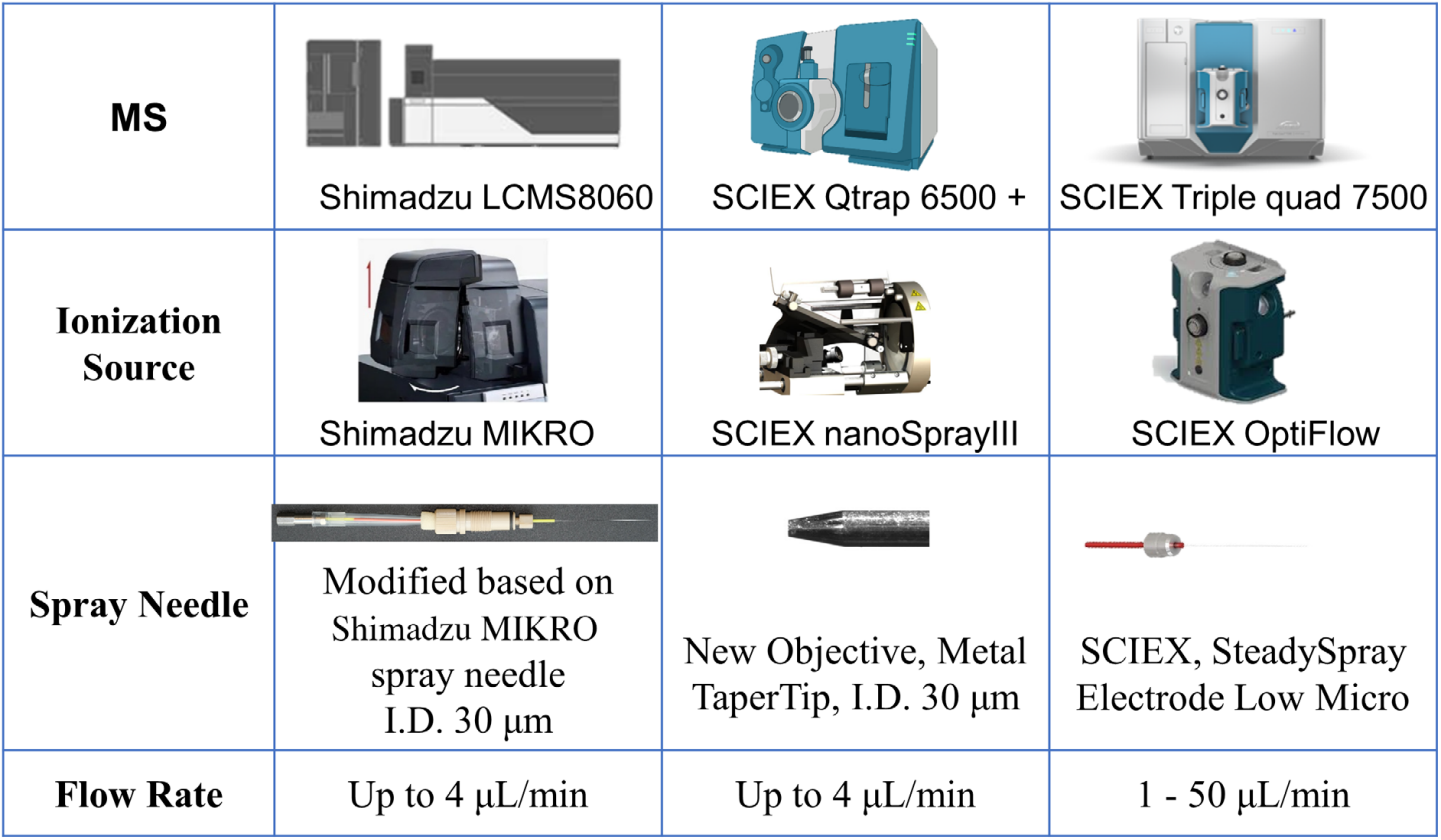
Mass spectrometers, ionization sources, and micro‐flow spray needles used for performance comparison.

**FIGURE 2:**
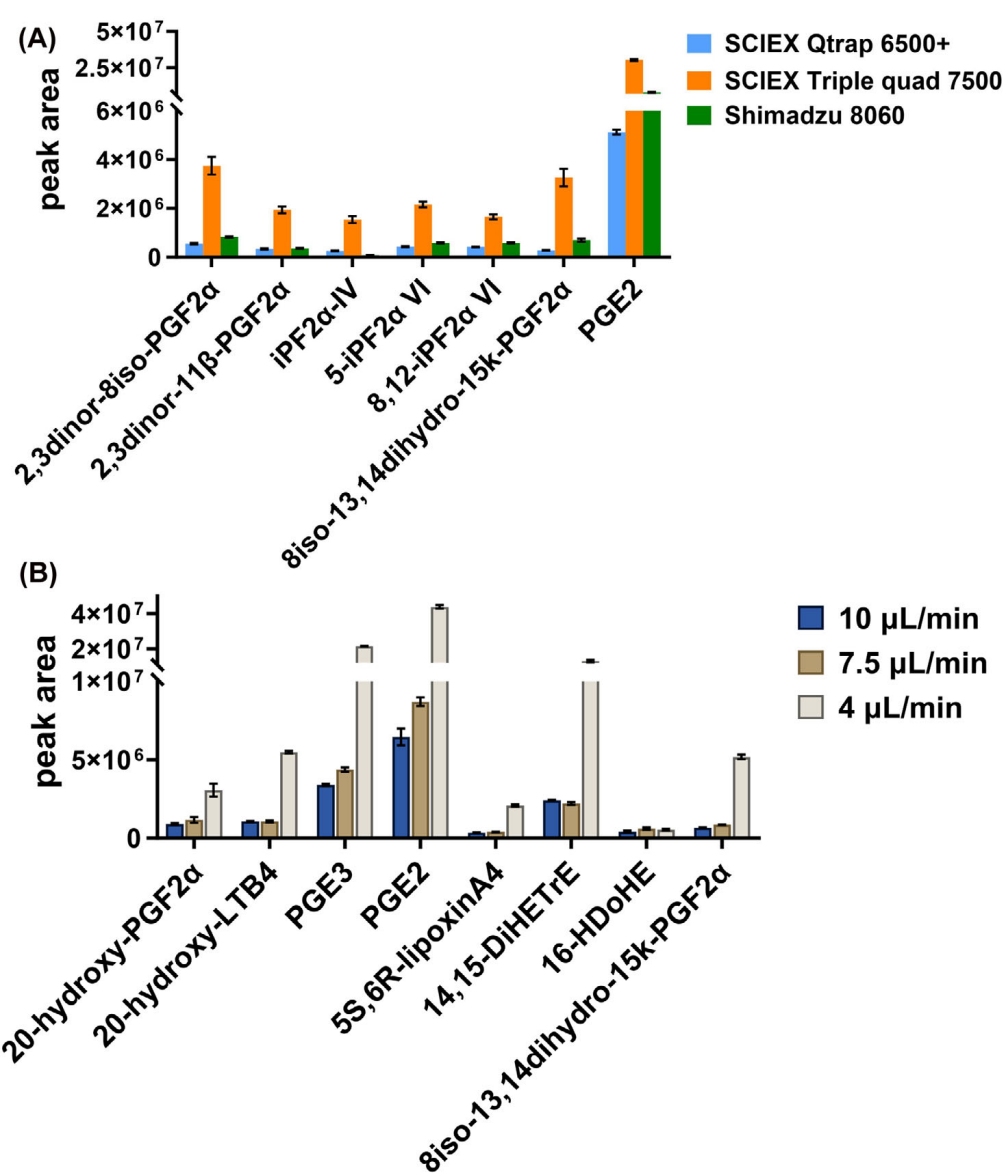
(A) Comparison of the performance from different MS systems using 4 µL/min; (B) optimization of micro‐flow rate on SCIEX triple quad 7500 MS.

Significant sensitivity enhancement was achieved by employing a nanoAcquity LC coupled with Shimadzu 8060 MS and a homemade spray emitter under micro‐level flow rate as indicated in previous study [19]. Negative mode was used for the analysis of oxylipins. However, applying a negative voltage resulted in damage to the tip of the spray emitter due to severe corona discharge. Although the discharge problem could be mitigated by moving the emitter tip away from the MS orifice, sensitivity was sacrificed in this process. To address the discharge issue while maintaining high sensitivity under micro‐flow rates, we compared several MS systems and micro‐flow ionization sources, including SCIEX NanoSpray III with a Metal TaperTips spray emitter, Shimadzu MIKRO ionization source with a homemade spray needle, and SCIEX OptiFlow with a SteadySpray electrode (Figure 1). All MS parameters were optimized for the optimal performance of each system. On the basis of its advantages in robustness and sensitivity (Figure 2A), a SCIEX triple quad 7500 MS with the Optiflow ionization source was ultimately selected for further method development. The SteadySpray needle also provided more flexibility with its tolerance of a wide range of flow rates.

To enhance the sensitivity of targeted compounds, we initially compared several flow rates. Figure 2B illustrates that the peak area of selected compounds increased with lower flow rate. It is also predictable that with 4 µL/min flow rate, the dilution factor of injected samples is lower. Simultaneously, a lower flow rate facilitates more efficient ionization of target compounds.

In order to improve the separation of isomers with same SRM transitions, including 8‐iso‐PGE2, PGE2, 11β‐PGE2, PGD2 (Q1/Q3 351.1/271.2), and 8iso‐15R‐PGF2α, 8iso‐PGF2α, 11β‐PGF2α, PGF2α (Q1/Q3 353.1/193.1), a 15‐cm length Phenomenex Kinetex C18 column was used. In addition, a 5‐min isocratic time at the beginning of the gradient with 80% mobile phase A was applied. This step is also crucial to prevent peak tailing in early eluting compounds, such as 20‐hydroxy‐PGE2 and 20‐hydroxy‐PGF2α (Figure S1).

**FIGURE 3:**
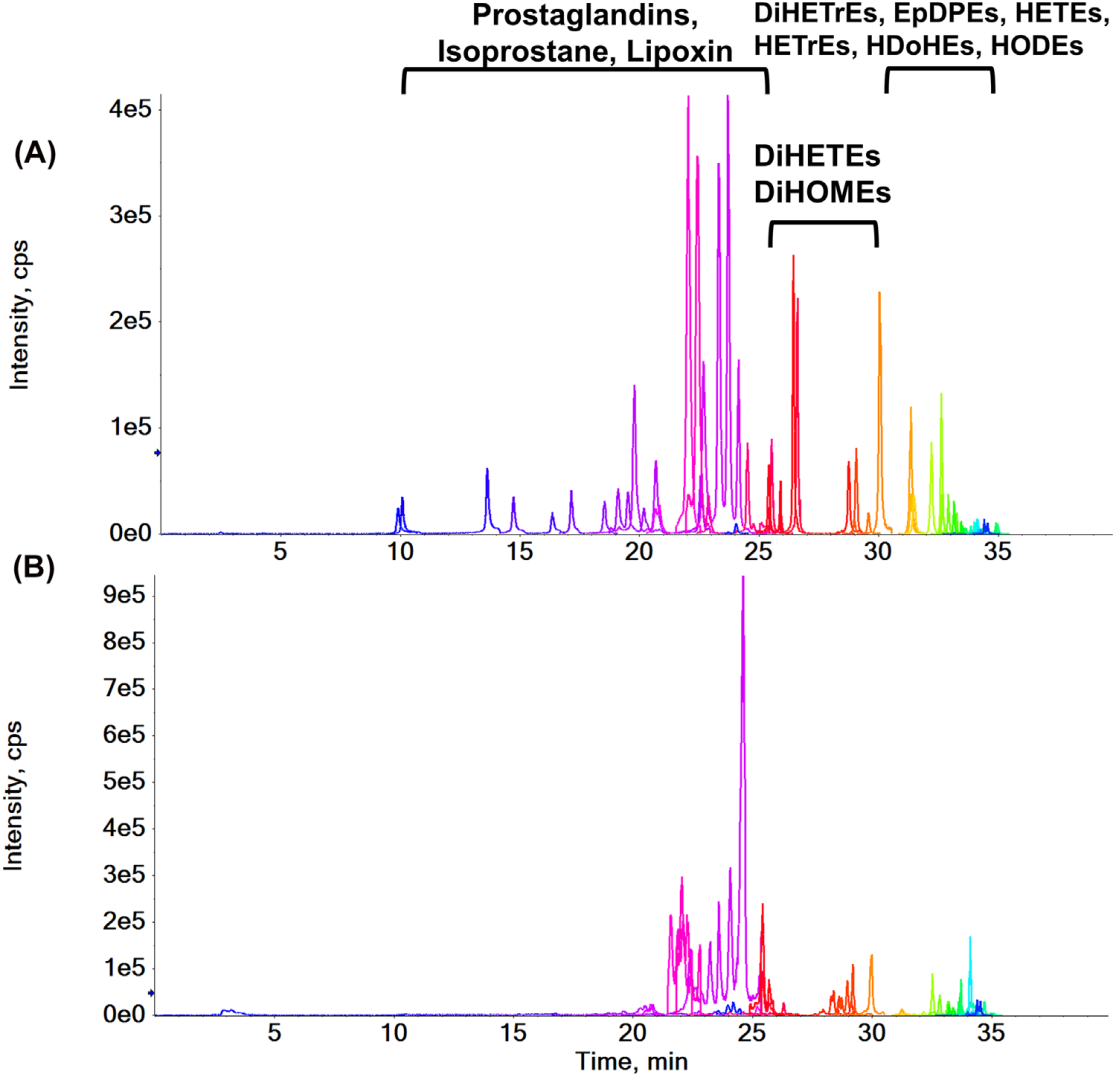
Overlay of SRM chromatograms of oxylipins obtained with the injection of (A) standard solution C7 and (B) 5 µL pooled plasma.

Following the optimization of micro‐LC–MS/MS conditions, all targeted compounds exhibited excellent resolution using a Phenomenex Kinetex C18 (2.6 µm, 0.3 × 150 mm^2^) column with a 40‐min analysis time. The injection volume was set at 3 µL. Typical SRM chromatograms of targeted oxylipins in standard solution and pooled human plasma are shown in Figure 3. Prostaglandins (PGs), isoprostane, and lipoxins eluted before 30 min, whereas dihydroxyeicosatrienoic acids (DiHETrEs), epoxy‐docosapentaenoic acids (EpDPEs), hydroxy‐eicosatrienoic acids (HETrEs), and hydroxyeicosatetraenoic acids (HETEs) with lower polarity eluted between 30 and 35 min. The optimized condition ensures robust analysis of the diverse oxylipin classes.

### 3.2 Sensitivity Enhancement Compared to a UHPLC–MS/MS Method

**FIGURE 4:**
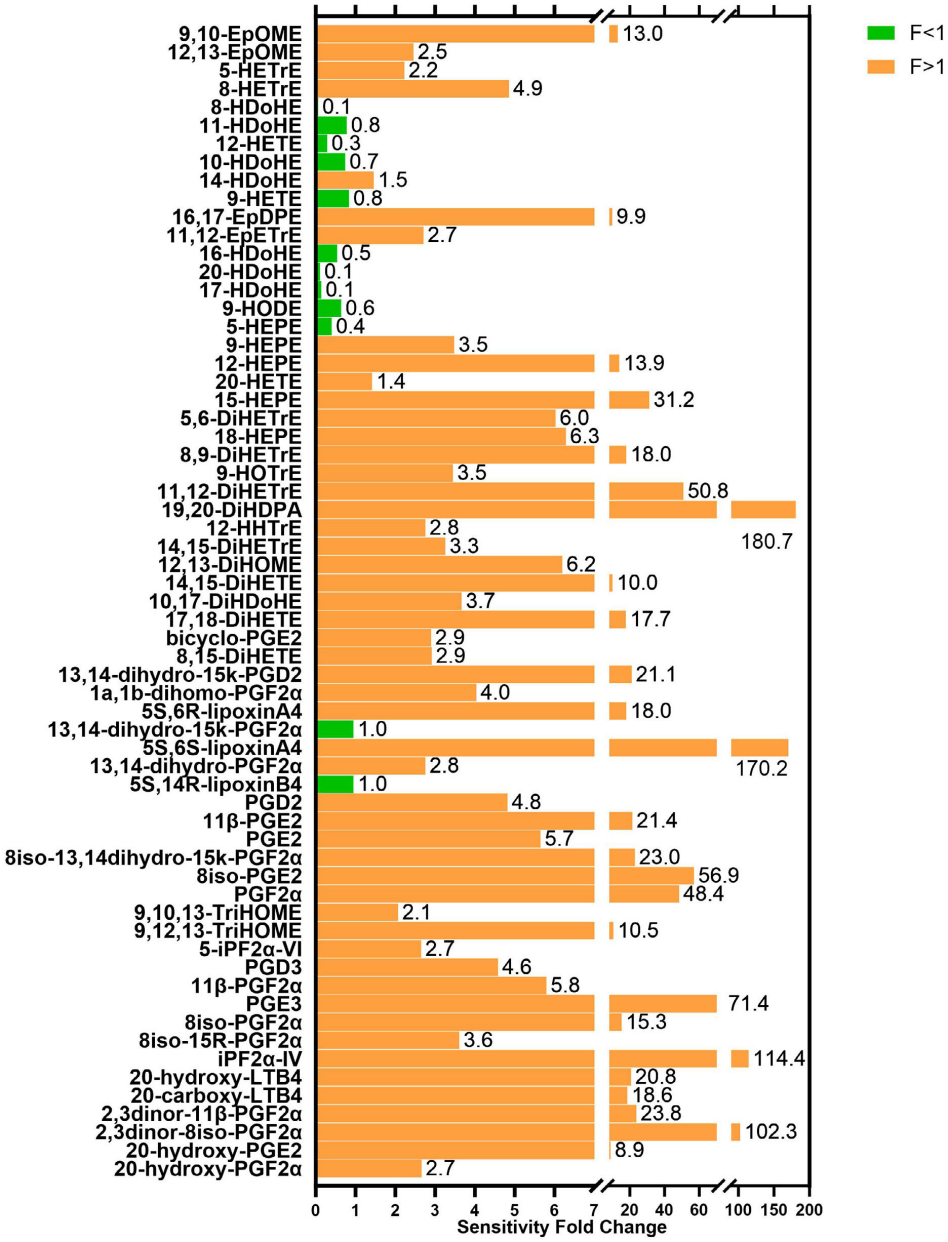
Sensitivity fold change (F) calculated by LOD (LOQ) of micro‐LC–MS/MS divided by LOD (LOQ) from UHPLC–MS/MS, compounds arranged in elution order from bottom to top.

The sensitivity enhancement of micro‐LC–MS/MS method was evaluated by comparing its LODs and LOQs with those of conventional UHPLC–MS/MS using calibration solutions. An extracted sample of 5 µL was injected on a Waters Acquity BEH C18 column (50 × 2.1 mm^2^, 1.7 µm) for conventional method, and 3 µL was injected on a Phenomenex Kinetex C18 column (2.6 µm, 150 × 0.3 mm^2^) for micro‐LC–MS/MS method. The same MS and ionization source were utilized for a fair comparison. In Figure 4, the fold changes in LOD and LOQ for the micro‐flow method over the conventional method are depicted for targeted oxylipins. For 13‐HODE, 8,12‐iPF2α‐VI, and 13,14‐dihydro‐15keto‐PGE2, the calibration points within the same concentration ranges of the micro‐LC–MS method were insufficient for calculating LOD and LOQ compared with the UHPLC–MS/MS method.

Among the other 63 oxylipins, sensitivity increased from 1.4 to 180.7 times in 51 compounds (orange bars in Figure 4). However, due to differences in the compounds’ physical–chemical properties, for 10 compounds (depicted as green bars in Figure 4), including 9‐HETE, 11‐HDoHE, 10‐HDoHE, 9‐HODE, 16‐HDoHE, 5‐HEPE, 12‐HETE, 17‐HDoHE, 20‐HDoHE, and 8‐HDoHE, micro‐LC–MS performance was found to be inferior to that of the UHPLC–MS/MS method. Different reconstitution solvents, columns, and LC gradients were used for the micro‐LC–MS and UHPLC–MS methods, which resulted in a change in chromatographic selectivity and lead to changes in ion suppression for those compounds. The ion suppression subsequently resulted in higher LOD and a lower signal‐to‐noise ratio for micro‐LC–MS. For detailed LOD and LOQ values for both methods, refer to Table S5.

### 3.3 Method Validation

The optimized method outlined in Section 2.4 was validated with the evaluation of linearity, precision, recovery, and matrix effects. To cater to the demands of quantification in 5 µL human plasma, the calibration solutions were diluted to picomolar level near the detection limits.

The linearity of all compounds was established using calibrant solutions, yielding coefficient of determination (*R* ^2^) values ranging from 0.9930 to 0.9999. Back‐calculated concentrations of the calibration standards fell within ±15% (20% for LOQ) of the nominal values, indicating satisfactory linearity for all analytes. The instrument LOQs were determined to be between 0.3 and 306.2 pM, as summarized in Table 1.

**TABLE 1:** Validation parameters: calibration range, limits of detections (LODs), and limits of quantifications (LOQs) in standard solutions.

Compound name	Linearity range (nM)	*R* ^2^	LOD (pM)	LOQ (pM)
20‐hydroxy‐PGF2α	0.12–29.93	0.9976	1.9	6.2
20‐hydroxy‐PGE2	0.02–21.3	0.9986	0.3	1.0
2,3dinor‐8iso‐PGF2α	0.07–37.5	0.9996	0.3	1.1
20‐carboxy‐LTB4	0.08–20.63	0.9988	1.3	4.2
2,3dinor‐11β‐PGF2α	0.07–37.5	0.9989	1.2	4.1
20‐hydroxy‐LTB4	0.04–20.85	0.9987	0.8	2.6
iPF2α‐IV	0.07–37.5	0.9979	0.2	0.8
8iso‐15R‐PGF2α	0.07–37.5	0.9994	0.8	2.8
8iso‐PGF2α	0.07–37.5	0.9995	2.1	6.8
11β‐PGF2α	0.12–29.63	0.9989	1.1	3.8
PGF2α	0.04–37.5	0.9991	0.2	0.8
PGE3	0.04–37.5	0.9989	0.1	0.3
PGD3	0.04–37.5	0.9991	0.6	1.8
8iso‐PGE2	0.04–37.5	0.9994	0.1	0.3
PGE2	0.04–37.5	0.9988	0.9	2.9
11β‐PGE2	0.03–29.78	0.9995	0.3	0.9
PGD2	0.04–37.5	0.9990	0.4	1.3
5‐iPF2α ‐VI	0.15–37.5	0.9989	2.5	8.3
8,12‐iPF2α‐VI	1.17–37.5	0.9991	23.0	76.5
9,12,13‐TriHOME	0.12–30.11	0.9988	6.6	22.0
9,10,13‐TriHOME	0.06–30.11	0.9990	4.6	15.4
8iso‐13,14dihydro‐15keto‐PGF2α	0.15–37.5	0.9996	1.1	3.6
13,14dihydro‐15keto‐PGF2α	0.12–14.81	0.9984	2.5	8.5
13,14dihydro‐PGF2α	0.03–14.74	0.9987	3.5	11.7
13,14dihydro‐15keto‐PGE2	0.03–14.89	0.9996	0.3	0.9
13,14dihydro‐15keto‐PGD2	0.03–29.78	0.9993	0.3	1.1
5S,6R‐lipoxinA4	0.04–20.85	0.9993	1.0	3.4
5S,6S‐lipoxinA4	0.02–10.43	0.9987	0.1	0.3
1a,1b‐dihomo‐PGF2α	0.03–27.45	0.9993	0.6	2.1
bicyclo‐PGE2	0.03–31.43	0.9991	0.9	3.0
8,15‐DiHETE	0.33–20.93	0.9985	6.1	20.5
10,17‐DiHDoHE	0.16–21	0.9984	4.7	15.8
17,18‐DiHETE	0.12–30	0.9991	2.4	8.0
14,15‐DiHETE	0.03–30	0.9989	0.7	2.3
12,13‐DiHOME	0.16–41.4	0.9986	2.3	7.6
14,15‐DiHETrE	0.02–21.08	0.9988	0.9	3.0
12‐HHTrE	0.02–21	0.9989	4.4	14.7
19,20‐DiHDPA	0.02–20.85	0.9987	0.1	0.4
11,12‐DiHETrE	0.02–10.54	0.9991	0.1	0.4
9‐HOTrE	0.04–21.41	0.9982	2.6	8.8
8,9‐DiHETrE	0.02–21	0.9986	0.5	1.6
18‐HEPE	0.09–11.55	0.9975	3.0	9.9
15‐HEPE	0.04–21.15	0.9983	0.4	1.5
20‐HETE	0.08–21	0.9965	8.8	29.3
5,6‐DiHETrE	0.16–10.54	0.9973	2.2	7.4
12‐HEPE	0.17–21.15	0.9957	0.4	1.5
9‐HEPE	0.18–11.55	0.9948	3.9	12.9
13‐HODE	1.33–42.53	0.9981	59.0	196.6
12,13‐EpOME	0.16–20.55	0.9984	4.3	14.4
5‐HEPE	0.17–10.58	0.9978	5.7	19.1
9‐HODE	0.66–21.26	0.9981	27.7	92.3
9,10‐EpOME	0.08–20.55	0.9984	2.2	7.3
17‐HDoHE	1.34–21.38	0.9936	47.3	157.6
20‐HDoHE	1.34–21.38	0.9832	91.9	306.2
16‐HDoHE	0.33–21	0.9982	3.4	11.5
16,17‐EpDPE	0.17–10.69	0.9967	5.1	16.8
9‐HETE	0.48–30.53	0.9970	8.3	27.8
11,12‐EpETrE	0.33–21	0.9968	0.6	1.8
14‐HDoHE	0.04–5.25	0.9946	7.1	23.6
10‐HDoHE	0.33–21.38	0.9980	12.8	42.6
12‐HETE	0.17–21.38	0.9942	21.9	72.9
11‐HDoHE	0.66–21	0.9944	8.8	29.2
8‐HDoHE	0.33–21.38	0.9964	76.4	254.6
8‐HETrE	1.34–21.38	0.9981	4.4	14.5
5‐HETrE	0.18–22.8	0.9961	4.7	15.6
5S,14R‐lipoxinB4	0.18–22.8	0.9972	11.6	38.6

Intra‐ and interday precisions varied from 0.5% to 11.7% and from 2.5% to 14.1%, respectively. All values were within the 15% tolerance limit, underscoring the repeatability of the method, as detailed in Table 2.

**TABLE 2:** Intra‐ and interday precisions (RSD%) of oxylipins spiked to plasma.

Compounds	Intraday precision (%)			Interday precision (%)		
	Low	Medium	High	Low	Medium	High
d_4_‐8iso‐PGF2α	11.4	9.6	5.2	5.8	6.0	3.5
d_4_‐PGF2α	14.1	3.6	6.3	2.3	4.9	2.8
d_11_‐5‐iPF2α‐VI	9.6	8.0	6.2	2.9	2.5	5.5
d_11_‐8,12‐iso‐iPF2α‐VI	2.5	4.5	3.5	2.9	4.2	3.6
d_4_‐8iso‐PGE2	5.8	2.9	3.7	2.0	1.2	1.7
d_4_‐PGE2	14.1	3.6	4.6	7.4	4.0	3.4
d_4_‐PGD2	7.1	6.5	6.9	1.5	2.1	2.9
d_9_‐PGE2	2.5	2.6	5.9	1.0	1.4	0.7
d_4_‐LTB4	7.1	5.3	9.0	1.6	1.4	0.7
d_4_‐12,13‐DiHOME	8.7	6.0	9.0	11.7	2.8	4.3
d_4_‐9,10‐DiHOME	5.8	6.6	2.6	1.7	1.3	1.8
d_11_‐14,15‐DiHETrE	4.3	8.7	7.4	0.5	3.3	2.3
d_6_‐20‐HETE	13.5	6.8	12.1	9.8	10.0	12.5

**FIGURE 5:**
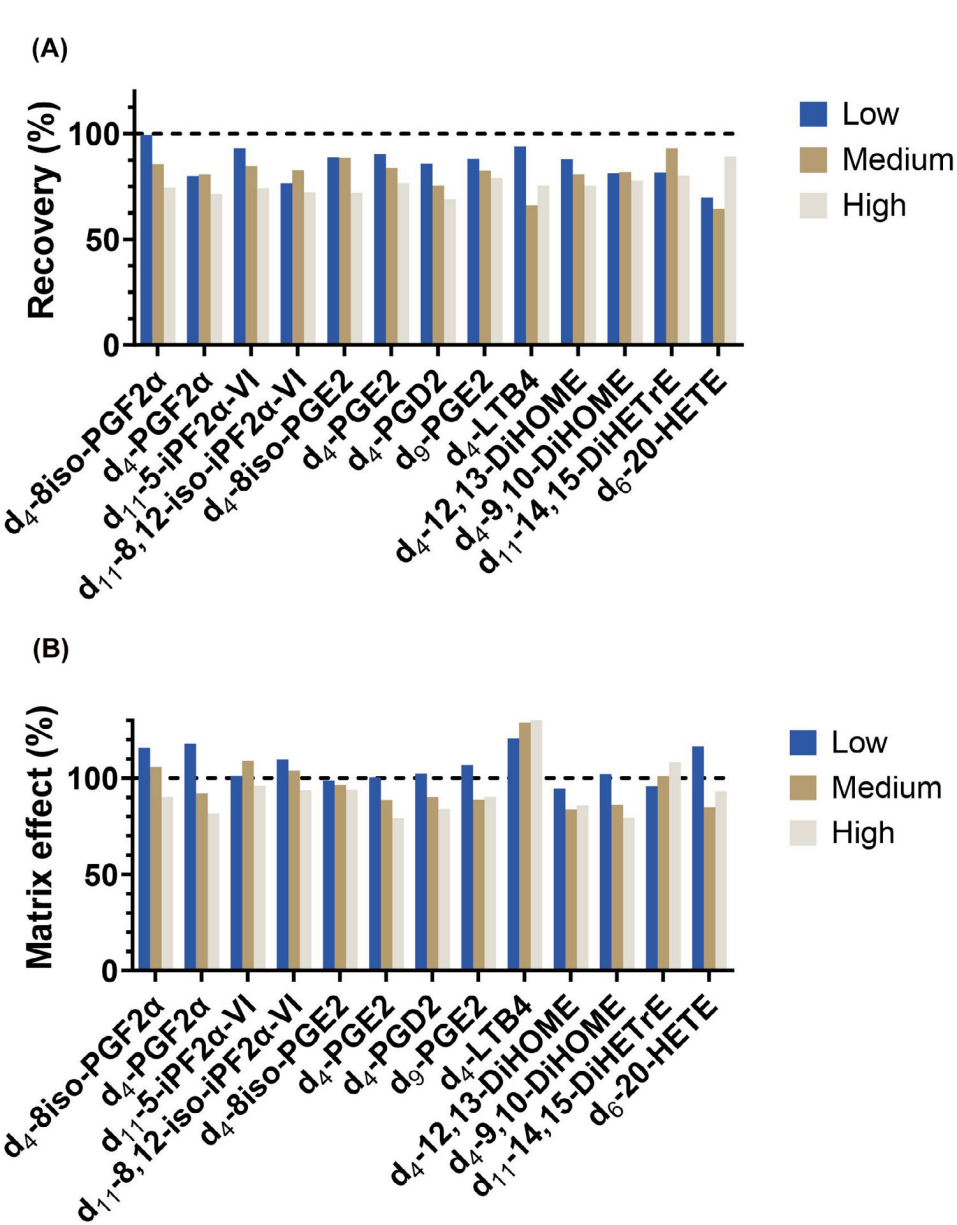
Recovery and matrix effect of deuterated internal standards in human plasma. Recovery and matrix effect values are expressed in percentages. (A) Recovery: higher values indicate better recoveries. (B) Matrix effect: values above 100% indicate ion enhancement and below 100% imply ion suppression.

Matrix effect suggests ion enhancement or suppression during MS ionization due to matrix components. Matrix effects above 100% indicate ion enhancement, suggesting that components in the matrix positively influence the ionization of analytes, leading to higher signal intensities. Conversely, matrix effects below 100% suggest ion suppression, where the matrix components hinder the ionization of analytes, resulting in reduced signal intensities. Deuterated ISTDs were employed for the determination of recovery and matrix effect. The recoveries ranged from 64.6% to 99.6%. Matrix effect ranged from 79.2% to 130.3% (Figure 5). Particularly, ion enhancement was observed in LTB4‐d_4_. All compounds demonstrated acceptable recovery rates and consistent matrix effects across three concentration levels, affirming the applicability of the sample preparation method for quantitative analysis. The results collectively emphasize the robustness and reliability of the validated method for the sensitive quantification of oxylipins in volume‐limited human plasma.

### 3.4 Application in a Sample‐Limited Clinical Study Modeling Healthy Aging

The validated method was applied to a healthy aging study involving 40 human plasma samples grouped by genders. Three samples were excluded due to technical errors. Four pooled plasma samples were analyzed along with the study samples as QC samples. Compounds with >80% missing values in study samples or >50% missing values in QCs were also excluded for data reliability. As presented in Table 3, a total of 28 oxylipins were successfully quantified in study samples, revealing a broad concentration range spanning from 51.8 ± 21.8 pM (8,9‐DiHETrE in male samples) to 30.9 ± 8.9 nM (13‐HODE). These findings underscore the applicability of the developed micro‐LC–MS/MS method in analyzing oxylipins within volume‐limited plasma samples, particularly in the context of studies focused on healthy aging. It is worth noting that the current results serve as a promising indication of the method's suitability for oxylipin studies in volume‐limited plasma, given the diverse range of concentrations successfully determined.

**TABLE 3:** Average concentrations of oxylipins in two groups of study samples (mean ± SD).

Oxylipins	Female (*n* = 18, pM)	Male(*n* = 19, pM)
5‐iPFα‐VI	152 ± 73	153 ± 80
8,12‐iso‐iPF2α‐VI	4115 ± 1930	4258 ± 1920
9,12,13‐TriHOME	1241 ± 737	2297 ± 1725
9,10,13‐TriHOME	230 ± 184	227 ± 102
17,18‐DiHETE	2014 ± 896	1922 ± 765
14,15‐DiHETE	267 ± 121	243 ± 81
12,13‐DiHOME	3373 ± 1900	3845 ± 1816
14,15‐DiHETrE	148 ± 49	147 ± 30
12‐HHTrE	71 ± 20	206 ± 319
19,20‐DiHDPA	468 ± 182	473 ± 252
11,12‐DiHETrE	124 ± 41	120 ± 31
9‐HOTrE	1052 ± 638	850 ± 407
8,9‐DiHETrE	64 ± 26	52 ± 22
18‐HEPE	135 ± 63	127 ± 61
15‐HEPE	148 ± 91	119 ± 29
20‐HETE	306 ± 154	243 ± 90
5,6‐DiHETrE	196 ± 80	198 ± 107
12‐HEPE	71 ± 41	100 ± 84
13‐HODE	30852 ± 8892	29356 ± 11859
12,13‐EpOME	2239 ± 1534	2011 ± 806
5‐HEPE	302 ± 346	317 ± 137
9‐HODE	20316 ± 7779	23773 ± 13832
9,10‐EpOME	475 ± 314	427 ± 179
20‐HDoHE	779 ± 624	543 ± 278
16‐HDoHE	191 ± 218	142 ± 61
16,17‐EpDPE	341 ± 135	353 ± 169
11,12‐EpETrE	170 ± 55	181 ± 73
8‐HETrE	181 ± 69	162 ± 65

## 4 Conclusion

In this study, we developed a micro‐LC–MS/MS method tailored for the precise determination of oxylipins in volume‐limited human plasma. Requiring only 5 µL plasma, the method achieved LODs ranging from 0.1 to 91.9 pM, and LOQs ranging from 0.3 to 306.2 pM. The method exhibited good repeatability, with intra‐ and interday precisions consistently below 14.1%. To assess its sensitivity enhancement, the developed micro‐LC–MS/MS method underwent a thorough comparison with a conventional flow UHPLC–MS/MS method. The results demonstrated that our method is not only sensitive but also well suited for bioanalysis in volume‐limited plasma. We subsequently applied the validated method to analyze 40 human plasma samples for oxylipin determination. Although the difference between the two gender groups is not significant among the current sample set, the method is robust and could contribute to cohort studies with volume‐limited samples. With the wide coverage of concentration levels in different oxylipins and robustness for plasma analysis, our method is instrumental in its ability for future research in oxylipins, especially when confronted with volume‐limited samples. To pave the way for its broader utility in clinical and research settings, further development of the method should prioritize expanding the targets to other signaling lipids, such as fatty acids and bile acids, and evaluating its performance in extensive sample cohorts under diverse health conditions.

## Conflicts of Interest

The authors declare no conflicts of interest.

## Supporting information



Supporting Information

## Data Availability

The data that support the findings of this study are available on request from the corresponding author. The data are not publicly available due to privacy or ethical restrictions.
