# Supplementary material for: A Micro‐Flow Liquid Chromatography–Mass Spectrometry Method for the Quantification of Oxylipins in Volume‐Limited Human Plasma
Source: Electrophoresis. 2024 Nov 11;46(5-6):305–15. doi: 10.1002/elps.202400151 (PMC11952279; doi:10.1002/elps.202400151)
Supplement: Supplementary file 1 — Supporting Information [file ELPS-46--s001.docx]

Supporting Information

**A micro-flow liquid chromatography-mass spectrometry method for the quantification of oxylipins in volume-limited human plasma**

Bingshu He^1^, Rawi Ramautar^1^, Marian Beekman^2^, P. Eline Slagboom^2^, Amy Harms^1*^, Thomas Hankemeier^1*^

^1^Metabolomics and Analytics Centre, Leiden Academic Centre for Drug Research (LACDR), Leiden University, Leiden, The Netherlands.

^2^Department of Molecular Epidemiology, Leiden University Medical Centre, Leiden, The Netherlands

*Correspondence should be addressed to the following author:

Dr. Amy Harms

Metabolomics and Analytics Centre, Leiden Academic Centre for Drug Research (LACDR)

Leiden University

Einsteinweg 55, 2333 CC, Leiden, The Netherlands

Email a.c.harms@lacdr.leidenuniv.nl

Prof. Thomas Hankemeier

Metabolomics and Analytics Centre, Leiden Academic Centre for Drug Research (LACDR)

Leiden University

Einsteinweg 55, 2333 CC, Leiden, The Netherlands

Email hankemeier@lacdr.leidenuniv.nl

**Supplementary Table 1.** Abbreviations and purchasing information of oxylipins and labeled standards

| Abbr. | Common Name | Systematic name | Molecular formula | Cayman | CAS | Mass | HMDB |
| --- | --- | --- | --- | --- | --- | --- | --- |
| 20-hydroxy-PGF2α | 20-hydroxy Prostaglandin F2α | 9α,11α,15S,20-tetrahydroxy-prosta-5Z,13E-dien-1-oic acid | C_20_H_34_O_6_ | 16950 | 57930-92-4 | 370.2355 | HMDB0004049 |
| 20-hydroxy-PGE2 | 20-Hydroxy-PGE2 | 9-oxo-11α,15S,20-trihydroxy-prosta-5Z,13E-dien-1-oic acid | C_20_H_32_O_6_ | 14950 | 57930-95-7 | 368.2199 | HMDB0003247 |
| 2,3-dinor-8-iso-PGF2α | 2,3-dinor-8-iso Prostaglandin F2α | 9α,11α,15S-trihydroxy-2,3-dinor-(8β)-prosta-5Z,13E-dien-1-oic acid | C_18_H_30_O_5_ | 16290 | 221664-05-7 | 326.4000 | NA |
| 20-carboxy-LTB4 | 20-Carboxy-leukotriene B4 | 5S,12R-dihydroxy-6Z,8E,10E,14Z-eicosatetraene-1,20-dioic acid | C_20_H_30_O_6_ | 20180 | 80434-82-8 | 366.2042 | HMDB0006059 |
| 2,3-dinor-11β-PGF2α | 2,3-dinor-11β-Prostaglandin F2α | 9α,11β,15S-trihydroxy-2,3-dinor-prosta-5Z,13E-dien-1-oic acid | C_18_H_30_O_5_ | 16530 | 240405-20-3 | 326.4000 | NA |
| 20-hydroxy-LTB4 | 20-Hydroxy-leukotriene B4 | 5S,12R,20-trihydroxy-6Z,8E,10E,14Z-eicosatetraenoic acid | C_20_H_32_O_5_ | 20190 | 79516-82-8 | 352.2249 | HMDB0001509 |
| iPF2α-IV | iPF2alpha-IV | (8S)-10-[(1R,2S,3S,5R)-3,5-Dihydroxy-2-pentylcyclopentyl]-8-hydroxydeca-5,9-dienoic acid | C_20_H_34_O_5_ |  | 331962-00-6 | 354.2000 | NA |
| 8-iso-15-R-PGF2α | 8-iso-15(R)-Prostaglandin F2α | 9α,11α,15R-trihydroxy-(8β)-prosta-5Z,13E-dien-1-oic acid | C_20_H_34_O_5_ | 16395 | 214748-65-9 | 354.2406 | NA |
| 8-iso-PGF2a | 8-iso-15(S)-Prostaglandin F2α | 9α,11α,15S-trihydroxy-(8β)-prosta-5Z,13E-dien-1-oic acid | C_20_H_34_O_5_ | 16350 | 27415-26-5 | 354.2406 | HMDB0005083 |
| 11β-PGF2a | 11β-Prostaglandin F2α | 9α,11β,15S-trihydroxy-prosta-5Z,13E-dien-1-oic acid | C_20_H_34_O_5_ | 16520 | 38432-87-0 | 354.2406 | NA |
| PGF2α | Prostaglandin F2α | 9α,11α,15S-trihydroxy-prosta-5Z,13E-dien-1-oic acid | C_20_H_34_O_5_ | 16010 | 551-11-1 | 354.2406 | HMDB0001139 |
| PGE3 | Prostaglandin E3 | 9-oxo-11α,15S-dihydroxy-prosta-5Z,13E,17Z-trien-1-oic acid | C_20_H_30_O_5_ | 14990 | 802-31-3 | 350.2093 | HMDB0002664 |
| PGD3 | Prostaglandin D3 | 9α,15S-dihydroxy-11-oxo-prosta-5Z,13E,17Z-trien-1-oic acid | C_20_H_30_O_5_ | 12990 | 71902-47-1 | 350.2093 | HMDB0003034 |
| 8-iso-PGE2 | 8-iso Prostaglandin E2 | 9-oxo-11α,15S-dihydroxy-(8β)-prosta-5Z,13E-dien-1-oic acid | C_20_H_32_O_5_ | 14350 | 27415-25-4 | 352.2250 | HMDB0005844 |
| PGE2 | Prostaglandin E2 | 9-oxo-11α,15S-dihydroxy-prosta-5Z,13E-dien-1-oic acid | C_20_H_32_O_5_ | 14010 | 363-24-6 | 352.2250 | HMDB0001220 |
| 11β-PGE2 | 11β-Prostaglandin E2 | 9-oxo-11β,15S-dihydroxy-prosta-5Z,13E-dien-1-oic acid | C_20_H_32_O_5_ | 14510 | 38310-90-6 | 352.2250 | HMDB0060041 |
| PGD2 | Prostaglandin D2 | 9α,15S-dihydroxy-11-oxo-prosta-5Z,13E-dien-1-oic acid | C_20_H_32_O_5_ | 12010 | 41598-07-6 | 352.2250 | HMDB0001403 |
| 5-iPF2α VI | (±)5-iPF2α-VI | (8β)-5,9α,11α-trihydroxy-prosta-6E,14Z-dien-1-oic acid | C_20_H_34_O_5_ | 16300 | 179094-11-2 | 354.2406 | NA |
| 8,12-iPF2α VI | 8,12-iso-iPF2α-VI-1,5-lactone | 6-((E)-2-((1R,2S,3R,5S)-3,5-dihydroxy-2-((Z)-oct-2-enyl)cyclopentyl)vinyl)tetrahydro-2H-pyran-2-one | C_20_H_32_O_4_ | 10312 |  | 336.5000 | NA |
| 9,12,13-TriHOME | 9(S),12(S),13(S)-TriHOME | 9S,12S,13S-trihydroxy-11E-octadecenoic acid | C_18_H_34_O_5_ | 10005143 | 97134-11-7 | 330.2406 | HMDB0004708 |
| 9,10,13-TriHOME | 9(S),10(S),13(S)-TriHOME | 9S,10S,13S-trihydroxy-11E-octadecenoic acid | C_18_H_34_O_5_ | 26768 | 135214-44-7 | 330.2406 |  |
| 8-iso-13,14-dihydro-15-keto-PGF2α | 8-iso-13,14-dihydro-15-keto Prostaglandin F2α | 9α,11α-dihydroxy-15-oxo-(8β)-prost-5Z-en-1-oic acid | C_20_H_34_O_5_ | 16380 | 191919-02-5 | 354.2406 | HMDB0006562 |
| 13,14-dihydro-15-keto-PGF2α | 13,14-dihydro-15-keto Prostaglandin F2α | 9α,11α-dihydroxy-15-oxo-prost-5Z-en-1-oic acid | C_20_H_34_O_5_ | 16670 | 27376-76-7 | 354.2406 | HMDB0004685 |
| 13,14-dihydro-PGF2α | 13,14-dihydro Prostaglandin F2α | 9α,11α,15S-trihydroxy-prost-5Z-en-1-oic acid | C_20_H_36_O_5_ | 16660 | 27376-74-5 | 356.2563 | HMDB0004239 |
| 13,14-dihydro-15-keto-PGE2 | 13,14-dihydro-15-keto Prostaglandin E2 | 9,15-dioxo-11α-hydroxy-prost-5Z-en-1-oic acid | C_20_H_32_O_5_ | 14650 | 363-23-5 | 352.2250 | HMDB0002776 |
| 13,14-dihydro-15-keto-PGD2 | 13,14-dihydro-15-keto Prostaglandin D2 | 9α-hydroxy-11,15-dioxo-prost-5Z-en-1-oic acid | C_20_H_32_O_5_ | 12610 | 59894-07-4 | 352.2250 | HMDB0060042 |
| 5S,6R-lipoxinA4 | Lipoxin A4 | 5S,6R,15S-trihydroxy-7E,9E,11Z,13E-eicosatetraenoic acid | C_20_H_32_O_5_ | 90410 | 89663-86-5 | 352.2250 | HMDB0004385 |
| 5S,6S-lipoxinA4 | 6(S)-Lipoxin A4 | 5S,6S,15S-trihydroxy-7E,9E,11Z,13E-eicosatetraenoic acid | C_20_H_32_O_5_ | 10049 | 94292-80-5 | 352.2250 | NA |
| 1a,1b-dihomo-PGF2α | 1a,1b-dihomo Prostaglandin F2α | 9α,11α,15S-trihydroxy-1a,1b-dihomo-prosta-5Z,13E-dien-1-oic acid | C_22_H_38_O_5_ | 16050 | 57944-39-5 | 382.2719 | NA |
| bicyclo-PGE2 | Bicyclo Prostaglandin E2 | 11-deoxy-13,14-dihydro-15-keto-11β,16.xi.-cycloprostaglandin E2 | C_20_H_30_O_4_ | 14530 | 74158-09-1 | 334.2144 | HMDB0060054 |
| 8,15-DiHETE | 8(S),15(S)-DiHETE | 8S,15S-dihydroxy-5Z,9E,11Z,13E-eicosatetraenoic acid | C_20_H_32_O_4_ | 35370 | 80234-65-7 | 336.5000 | NA |
| 10,17-DiHDoHE | 10(S),17(S)-DiHDHA | 10(S),17(S)-dihydroxy-4Z,7Z,11E,13Z,15E,19Z-docosahexaenoic acid | C_22_H_32_O_4_ | 10008128 | 871826-47-0 | 360.2301 | NA |
| 17,18-DiHETE | (±)17(18)-DiHETE | (±)17,18-dihydroxy-5Z,8Z,11Z,14Z-eicosatetraenoic acid | C_20_H_32_O_4_ | 10006999 |  | 336.2301 | HMDB0010211 |
| 14,15-DiHETE | (±)14(15)-DiHETE | (±)14,15-dihydroxy-5Z,8Z,11Z,17Z-eicosatetraenoic acid | C_20_H_32_O_4_ | 10006998 |  | 336.2301 | HMDB0010204 |
| 12,13-DiHOME | (±)12(13)-DiHOME | (±)12,13-dihydroxy-9Z-octadecenoic acid | C_18_H_34_O_4_ | 10009832 | 263399-35-5 | 314.2457 | HMDB0004705 |
| 14,15-DiHETrE | (±)14(15)-DiHET | (±)14,15-dihydroxy-5Z,8Z,11Z-eicosatrienoic acid | C_20_H_34_O_4_ | 51651 | 77667-09-5 | 338.2457 | HMDB0002265 |
| 12-HHTrE | 12(S)-HHTrE | 12S-hydroxy-5Z,8E,10E-heptadecatrienoic acid | C_17_H_28_O_3_ | 34590 | 54397-84-1 | 280.2038 | HMDB0012535 |
| 19,20-DiHDPA | (±)19(20)-DiHDPA | (±)19,20-dihydroxy-4Z,7Z,10Z,13Z,16Z-docosapentaenoic acid | C_22_H_34_O_4_ | 10007001 |  | 362.2457 | HMDB0010214 |
| 11,12-DiHETrE | (±)11(12)-DiHET | (±)11,12-dihydroxy-5Z,8Z,14Z-eicosatrienoic acid | C_20_H_34_O_4_ | 51511 | 192461-95-3 | 338.2457 | HMDB0002314 |
| 9-HOTrE | 9(S)-HOTrE | 9S-hydroxy-10E,12Z,15Z-octadecatrienoic acid | C_18_H_30_O_3_ | 39420 | 89886-42-0 | 294.2195 | HMDB0031934 |
| 8,9-DiHETrE | (±)8(9)-DiHET | (±)8,9-dihydroxy-5Z,11Z,14Z-eicosatrienoic acid | C_20_H_34_O_4_ | 51351 | 192461-96-4 | 338.2457 | HMDB0002311 |
| 18-HEPE | (±)18-HEPE | (±)-18-hydroxy-5Z,8Z,11Z,14Z,16E-eicosapentaenoic acid | C_20_H_30_O_3_ | 32840 | 141110-17-0 | 318.2195 | HMDB0012611(R) |
| 15-HEPE | (±)15-HEPE | (±)-15-hydroxy-5Z,8Z,11Z,13E,17Z-eicosapentaenoic acid | C_20_H_30_O_3_ | 32700 | 88852-33-9 | 318.2195 | HMDB0010209 |
| 20-HETE | (±)20-HETE | 20-hydroxy-5Z,8Z,11Z,14Z-eicosatetraenoic acid | C_20_H_32_O_3_ | 90030 | 79551-86-3 | 320.2351 | HMDB0005998 |
| 5,6-DiHETrE | (±)5(6)-DiHET | (±)5,6-dihydroxy-8Z,11Z,14Z-eicosatrienoic acid | C_20_H_34_O_4_ | 51211 | 213382-49-1 | 338.2457 | HMDB0002343 |
| 12-HEPE | (±)12-HEPE | (±)-12-hydroxy-5Z,8Z,10E,14Z,17Z-eicosapentaenoic acid | C_20_H_30_O_3_ | 32540 | 81187-21-5 | 318.2195 | HMDB10202 |
| 9-HEPE | (±)9-HEPE | (±)-9-hydroxy-5Z,7E,11Z,14Z,17Z-eicosapentaenoic acid | C_20_H_30_O_3_ | 32400 | 286390-03-2 | 318.2195 | HMDB0060053 |
| 13-HODE | (±)13-HODE | (±)-13-hydroxy-9Z,11E-octadecadienoic acid | C_18_H_32_O_3_ | 38600 | 18104-45-5 | 296.2351 | HMDB0112194 |
| 12,13-EpOME | (±)12(13)-EpOME | (±)12(13)epoxy-9Z-octadecenoic acid | C_18_H_32_O_3_ | 52450 |  | 296.2351 | HMDB0004702 |
| 5-HEPE | (±)5-HEPE | (±)-5-hydroxy-6E,8Z,11Z,14Z,17Z-eicosapentaenoic acid | C_20_H_30_O_3_ | 32200 | 83952-40-3 | 318.2195 | HMDB0005081 |
| 9-HODE | (±)9-HODE | (±)-9-hydroxy-10E,12Z-octadecadienoic acid | C_18_H_32_O_3_ | 38400 | 98524-19-7 | 296.2351 | HMDB0010223 |
| 9,10-EpOME | (±)9(10)-EpOME | (±)9,10-epoxy-12Z-octadecenoic acid | C_18_H_32_O_3_ | 52400 |  | 296.2351 | HMDB0004701 |
| 17-HDoHE | (+/-)-17-HDoHE | (±)17-hydroxy-4Z,7Z,10Z,13Z,15E,19Z-docosahexaenoic acid | C_22_H_32_O_3_ | 33650 | 90780-52-2 | 344.2351 | HMDB0010213 |
| 20-HDoHE | (+/-) 20-HDoHE | (±)20-hydroxy-4Z,7Z,10Z,13Z,16Z,18E-docosahexaenoic acid | C_22_H_32_O_3_ | 33750 | 90906-41-5 | 344.2351 | HMDB0060048 |
| 16-HDoHE | (+/-) 16-HDoHE | (±)16-hydroxy-4Z,7Z,10Z,13Z,17E,19Z-docosahexaenoic acid | C_22_H_32_O_3_ | 33600 | 90780-51-1 | 344.2351 | HMDB0060047 |
| 16,17-EpDPE | (±)16(17)-EpDPA | (±)16,17-epoxy-4Z,7Z,10Z,13Z,19Z-docosapentaenoic acid | C_22_H_32_O_3_ | 10174 | 155073-46-4 | 344.2351 | HMDB0013621 |
| 9-HETE | (±)9-HETE | (±)-9-hydroxy-5Z,7E,11Z,14Z-eicosatetraenoic acid | C_20_H_32_O_3_ | 34400 | 79495-85-5 | 320.2351 | HMDB0010222 |
| 11,12-EpETrE | (±)11,12-EpETrE | (±)11,(12)-epoxy-5Z,8Z,14Z-eicosatrienoic acid | C_20_H_32_O_3_ | 50511 | 123931-40-8 | 320.2351 | HMDB0004673 |
| 14-HDoHE | (+/-) 14-HDoHE | (±)14-hydroxy-4Z,7Z,10Z,12E,16Z,19Z-docosahexaenoic acid | C_22_H_32_O_3_ | 33550 | 87042-40-8 | 344.2351 | HMDB0060044 |
| 10-HDoHE | (+/-) 10-HDoHE | (±)10-hydroxy-4Z,7Z,11E,13Z,16Z,19Z-docosahexaenoic acid | C_22_H_32_O_3_ | 33400 | 90780-50-0 | 344.2351 | HMDB0060037 |
| 12-HETE | (±)12-HETE | (±)12-hydroxy-5Z,8Z,10E,14Z-eicosatetraenoic acid | C_20_H_32_O_3_ | 34550 | 71030-37-0 | 320.2351 | HMDB0006111 |
| 11-HDoHE | (+/-) 11-HDoHE | (±)11-hydroxy-4Z,7Z,9E,13Z,16Z,19Z-docosahexaenoic acid | C_22_H_32_O_3_ |  |  | 344.2351 | HMDB0060040 |
| 8-HDoHE | (+/-) 8-HDoHE | (±)8-hydroxy-4Z,6E,10Z,13Z,16Z,19Z-docosahexaenoic acid | C_22_H_32_O_3_ | 33350 | 90780-54-4 | 344.2351 | HMDB0060051 |
| 8-HETrE | 8(S)-HETrE | 8S-hydroxy-9E,11Z,14Z-eicosatrienoic acid | C_20_H_34_O_3_ | 36360 | 889573-69-7 | 322.2508 | NA |
| 5-HETrE | 5(S)-HETrE | 5S-hydroxy-6E,8Z,11Z-eicosatrienoic acid | C_20_H_34_O_3_ | 36230 | 195061-94-0 | 322.2508 | NA |
| 5S-14R-lipoxin B4 | Lipoxin B4 | 5S,14R,15S-trihydroxy-6E,8Z,10E,12E-eicosatetraenoic acid | C_20_H_32_O_5_ | 90420 | 98049-69-5 | 352.2250 | HMDB0005082 |
| d_4_-8iso-PGF2α | 8-iso Prostaglandin F2α-d_4_ | 9α,11α,15S-trihydroxy-(8β)-prosta-5Z,13E-dien-1-oic-3,3,4,4-d4 acid | C_20_H_30_D_4_O_5_ | 316350 | 211105-40-7 | 358.48 |  |
| d_4_-PGF2α | Prostaglandin F2α-d_4_ | 9α,11α,15S-trihydroxy-prosta-5Z,13E-dien-1-oic-3,3,4,4-d4 acid | C_20_H_30_D_4_O_5_ | 316010 | 34210-11-2 | 358.48 |  |
| d_11_-5-iPF2α-VI | (±)5-iPF2α-VI-d_11_ | (±)5,9α,11α-trihydroxy-(8β)-prosta-6E,14Z-dien-1-oic-16,16,17,17,18,18,19,19,20,20,20-d11 acid | C_20_H_23_D_11_O_5_ | 10006654 | 936565-17-2 | 365.57 |  |
| d_11_-8,12-iso-iPF2α-VI | 8,12-iso-iPF2α-VI-d_11_ | (12α)-5,9α,11α-trihydroxy-prosta-6E,14Z-dien-1-oic-16,16,17,17,18,18,19,19,20,20,20-d11 acid | C_20_H_23_D_11_O_5_ | 10006878 | 1616977-85-5 | 365.6 |  |
| d_4_-8iso-PGE2 | 8-iso Prostaglandin E2-d_4_ | 9-oxo-11α,15S-dihydroxy-(8β)-prosta-5Z,13E-dien-1-oic-3,3,4,4-d4 acid | C_20_H_28_D_4_O_5_ | 10011321 | 34210-10-1 | 356.5 |  |
| d_4_-PGE2 | Prostaglandin E2-d_4_ | 9-oxo-11α,15S-dihydroxy-prosta-5Z,13E-dien-1-oic-3,3,4,4-d4 acid | C_20_H_28_D_4_O_5_ | 314010 | 1356347-42-6 | 361.5 |  |
| d_4_-PGD2 | Prostaglandin D2-d_4_ | 9α,15S-dihydroxy-11-oxo-prosta-5Z,13E-dien-1-oic-3,3,4,4-d4 acid | C_20_H_28_D_4_O_5_ | 312010 | 211105-29-2 | 356.5 |  |
| d_9_-PGE2 | Prostaglandin E2-d_9_ | 9-oxo-11α,15S-dihydroxy-prosta-5Z,13E-dien-1-oic-17,17,18,18,19,19,20,20,20-d9 acid | C_20_H_23_D_9_O_5_ | 10581 | 1356347-42-6 | 361.5 |  |
| d_4_-LTB4 | Leukotriene B4-d_4_ | 5S,12R-dihydroxy-6Z,8E,10E,14Z-eicosatetraenoic-6,7,14,15-d4 acid | C_20_H_28_D_4_O_4_ | 320110 | 124629-74-9 | 340.5 |  |
| d_4_-12,13-DiHOME | (±)12(13)-DiHOME-d_4_ | (±)12,13-dihydroxy-9Z-octadecenoic-9,10,12,13-d4 acid | C_18_H_30_D_4_O_4_ | 10009994 |  | 318.5 |  |
| d_4_-9,10-DiHOME | (±)9(10)-DiHOME-d_4_ | (±)9,10-dihydroxy-12Z-octadecenoic-9,10,12,13-d4 acid | C_18_H_30_D_4_O_4_ | 10009993 |  | 318.5 |  |
| d_11_-14,15-DiHETrE | (±)14(15)-DiHET-d_11_ | (±)14,15-dihydroxy-5Z,8Z,11Z-eicosatrienoic-16,16,17,17,18,18,19,19,20,20,20-d11 acid | C_20_H_23_D_11_O_4_ | 10008040 |  | 349.6 |  |
| d_6_-20-HETE | 20-HETE-d_6_ | 20-hydroxy-5Z,8Z,11Z,14Z-eicosatetraenoic-16,16,17,17,18,18-d6 acid | C_20_H_26_D_6_O_3_ | 390030 | 2548939-89-3 | 326.5 |  |

**Supplementary Table 2.** Calibrant concentrations of oxylipins

| **Calibration concentrations (pM)** | **C11** | **C10** | **C9** | **C8** | **C7** | **C6** | **C5** | **C4** | **C3** | **C2** | **C1** |
| --- | --- | --- | --- | --- | --- | --- | --- | --- | --- | --- | --- |
| 20-hydroxy-PGF2α | 29925.0 | 14962.5 | 7481.3 | 3740.6 | 1870.3 | 935.2 | 467.6 | 233.8 | 116.9 | 58.4 | 29.2 |
| 20-hydroxy-PGE2 | 21300.0 | 10650.0 | 5325.0 | 2662.5 | 1331.3 | 665.6 | 332.8 | 166.4 | 83.2 | 41.6 | 20.8 |
| 2,3dinor-8iso-PGF2α | 37500.0 | 18750.0 | 9375.0 | 4687.5 | 2343.8 | 1171.9 | 585.9 | 293.0 | 146.5 | 73.2 | 36.6 |
| 20-carboxy-LTB4 | 20625.0 | 10312.5 | 5156.3 | 2578.1 | 1289.1 | 644.5 | 322.3 | 161.1 | 80.6 | 40.3 | 20.1 |
| 2,3dinor-11β-PGF2α | 37500.0 | 18750.0 | 9375.0 | 4687.5 | 2343.8 | 1171.9 | 585.9 | 293.0 | 146.5 | 73.2 | 36.6 |
| 20-hydroxy-LTB4 | 20850.0 | 10425.0 | 5212.5 | 2606.3 | 1303.1 | 651.6 | 325.8 | 162.9 | 81.4 | 40.7 | 20.4 |
| iPF2α-IV | 37500.0 | 18750.0 | 9375.0 | 4687.5 | 2343.8 | 1171.9 | 585.9 | 293.0 | 146.5 | 73.2 | 36.6 |
| 8iso-15R-PGF2α | 37500.0 | 18750.0 | 9375.0 | 4687.5 | 2343.8 | 1171.9 | 585.9 | 293.0 | 146.5 | 73.2 | 36.6 |
| 8iso-PGF2α | 37500.0 | 18750.0 | 9375.0 | 4687.5 | 2343.8 | 1171.9 | 585.9 | 293.0 | 146.5 | 73.2 | 36.6 |
| 11β-PGF2α | 29625.0 | 14812.5 | 7406.3 | 3703.1 | 1851.6 | 925.8 | 462.9 | 231.4 | 115.7 | 57.9 | 28.9 |
| PGF2α | 37500.0 | 18750.0 | 9375.0 | 4687.5 | 2343.8 | 1171.9 | 585.9 | 293.0 | 146.5 | 73.2 | 36.6 |
| PGE3 | 37500.0 | 18750.0 | 9375.0 | 4687.5 | 2343.8 | 1171.9 | 585.9 | 293.0 | 146.5 | 73.2 | 36.6 |
| PGD3 | 37500.0 | 18750.0 | 9375.0 | 4687.5 | 2343.8 | 1171.9 | 585.9 | 293.0 | 146.5 | 73.2 | 36.6 |
| 8iso-PGE2 | 37500.0 | 18750.0 | 9375.0 | 4687.5 | 2343.8 | 1171.9 | 585.9 | 293.0 | 146.5 | 73.2 | 36.6 |
| PGE2 | 37500.0 | 18750.0 | 9375.0 | 4687.5 | 2343.8 | 1171.9 | 585.9 | 293.0 | 146.5 | 73.2 | 36.6 |
| 11β-PGE2 | 29775.0 | 14887.5 | 7443.8 | 3721.9 | 1860.9 | 930.5 | 465.2 | 232.6 | 116.3 | 58.2 | 29.1 |
| PGD2 | 37500.0 | 18750.0 | 9375.0 | 4687.5 | 2343.8 | 1171.9 | 585.9 | 293.0 | 146.5 | 73.2 | 36.6 |
| 5-iPF2α -VI | 37500.0 | 18750.0 | 9375.0 | 4687.5 | 2343.8 | 1171.9 | 585.9 | 293.0 | 146.5 | 73.2 | 36.6 |
| 8,12-iPF2α-VI | 37500.0 | 18750.0 | 9375.0 | 4687.5 | 2343.8 | 1171.9 | 585.9 | 293.0 | 146.5 | 73.2 | 36.6 |
| 9,12,13-TriHOME | 60225.0 | 30112.5 | 15056.3 | 7528.1 | 3764.1 | 1882.0 | 941.0 | 470.5 | 235.3 | 117.6 | 58.8 |
| 9,10,13-TriHOME | 60375.0 | 30187.5 | 15093.8 | 7546.9 | 3773.4 | 1886.7 | 943.4 | 471.7 | 235.8 | 117.9 | 59.0 |
| 8iso-13,14dihydro-15keto-PGF2α | 37500.0 | 18750.0 | 9375.0 | 4687.5 | 2343.8 | 1171.9 | 585.9 | 293.0 | 146.5 | 73.2 | 36.6 |
| 13,14dihydro-15keto-PGF2α | 29625.0 | 14812.5 | 7406.3 | 3703.1 | 1851.6 | 925.8 | 462.9 | 231.4 | 115.7 | 57.9 | 28.9 |
| 13,14dihydro-PGF2α | 29475.0 | 14737.5 | 7368.8 | 3684.4 | 1842.2 | 921.1 | 460.5 | 230.3 | 115.1 | 57.6 | 28.8 |
| 13,14dihydro-15keto-PGE2 | 29775.0 | 14887.5 | 7443.8 | 3721.9 | 1860.9 | 930.5 | 465.2 | 232.6 | 116.3 | 58.2 | 29.1 |
| 13,14dihydro-15keto-PGD2 | 29775.0 | 14887.5 | 7443.8 | 3721.9 | 1860.9 | 930.5 | 465.2 | 232.6 | 116.3 | 58.2 | 29.1 |
| 5S,6R-lipoxinA4 | 20850.0 | 10425.0 | 5212.5 | 2606.3 | 1303.1 | 651.6 | 325.8 | 162.9 | 81.4 | 40.7 | 20.4 |
| 5S,6S-lipoxinA4 | 20850.0 | 10425.0 | 5212.5 | 2606.3 | 1303.1 | 651.6 | 325.8 | 162.9 | 81.4 | 40.7 | 20.4 |
| 1a,1b-dihomo-PGF2α | 27450.0 | 13725.0 | 6862.5 | 3431.3 | 1715.6 | 857.8 | 428.9 | 214.5 | 107.2 | 53.6 | 26.8 |
| bicyclo-PGE2 | 31425.0 | 15712.5 | 7856.3 | 3928.1 | 1964.1 | 982.0 | 491.0 | 245.5 | 122.8 | 61.4 | 30.7 |
| 8,15-DiHETE | 20925.0 | 10462.5 | 5231.3 | 2615.6 | 1307.8 | 653.9 | 327.0 | 163.5 | 81.7 | 40.9 | 20.4 |
| 10,17-DiHDoHE | 21000.0 | 10500.0 | 5250.0 | 2625.0 | 1312.5 | 656.3 | 328.1 | 164.1 | 82.0 | 41.0 | 20.5 |
| 17,18-DiHETE | 30000.0 | 15000.0 | 7500.0 | 3750.0 | 1875.0 | 937.5 | 468.8 | 234.4 | 117.2 | 58.6 | 29.3 |
| 14,15-DiHETE | 30000.0 | 15000.0 | 7500.0 | 3750.0 | 1875.0 | 937.5 | 468.8 | 234.4 | 117.2 | 58.6 | 29.3 |
| 12,13-DiHOME | 41400.0 | 20700.0 | 10350.0 | 5175.0 | 2587.5 | 1293.8 | 646.9 | 323.4 | 161.7 | 80.9 | 40.4 |
| 14,15-DiHETrE | 21075.0 | 10537.5 | 5268.8 | 2634.4 | 1317.2 | 658.6 | 329.3 | 164.6 | 82.3 | 41.2 | 20.6 |
| 12-HHTrE | 21000.0 | 10500.0 | 5250.0 | 2625.0 | 1312.5 | 656.3 | 328.1 | 164.1 | 82.0 | 41.0 | 20.5 |
| 19,20-DiHDPA | 20850.0 | 10425.0 | 5212.5 | 2606.3 | 1303.1 | 651.6 | 325.8 | 162.9 | 81.4 | 40.7 | 20.4 |
| 11,12-DiHETrE | 21075.0 | 10537.5 | 5268.8 | 2634.4 | 1317.2 | 658.6 | 329.3 | 164.6 | 82.3 | 41.2 | 20.6 |
| 9-HOTrE | 42825.0 | 21412.5 | 10706.3 | 5353.1 | 2676.6 | 1338.3 | 669.1 | 334.6 | 167.3 | 83.6 | 41.8 |
| 8,9-DiHETrE | 21000.0 | 10500.0 | 5250.0 | 2625.0 | 1312.5 | 656.3 | 328.1 | 164.1 | 82.0 | 41.0 | 20.5 |
| 18-HEPE | 23100.0 | 11550.0 | 5775.0 | 2887.5 | 1443.8 | 721.9 | 360.9 | 180.5 | 90.2 | 45.1 | 22.6 |
| 15-HEPE | 21150.0 | 10575.0 | 5287.5 | 2643.8 | 1321.9 | 660.9 | 330.5 | 165.2 | 82.6 | 41.3 | 20.7 |
| 20-HETE | 21000.0 | 10500.0 | 5250.0 | 2625.0 | 1312.5 | 656.3 | 328.1 | 164.1 | 82.0 | 41.0 | 20.5 |
| 5,6-DiHETrE | 21075.0 | 10537.5 | 5268.8 | 2634.4 | 1317.2 | 658.6 | 329.3 | 164.6 | 82.3 | 41.2 | 20.6 |
| 12-HEPE | 21150.0 | 10575.0 | 5287.5 | 2643.8 | 1321.9 | 660.9 | 330.5 | 165.2 | 82.6 | 41.3 | 20.7 |
| 9-HEPE | 23100.0 | 11550.0 | 5775.0 | 2887.5 | 1443.8 | 721.9 | 360.9 | 180.5 | 90.2 | 45.1 | 22.6 |
| 13-HODE | 42525.0 | 21262.5 | 10631.3 | 5315.6 | 2657.8 | 1328.9 | 664.5 | 332.2 | 166.1 | 83.1 | 41.5 |
| 12,13-EpOME | 41100.0 | 20550.0 | 10275.0 | 5137.5 | 2568.8 | 1284.4 | 642.2 | 321.1 | 160.5 | 80.3 | 40.1 |
| 5-HEPE | 21150.0 | 10575.0 | 5287.5 | 2643.8 | 1321.9 | 660.9 | 330.5 | 165.2 | 82.6 | 41.3 | 20.7 |
| 9-HODE | 42525.0 | 21262.5 | 10631.3 | 5315.6 | 2657.8 | 1328.9 | 664.5 | 332.2 | 166.1 | 83.1 | 41.5 |
| 9,10-EpOME | 41100.0 | 20550.0 | 10275.0 | 5137.5 | 2568.8 | 1284.4 | 642.2 | 321.1 | 160.5 | 80.3 | 40.1 |
| 17-HDoHE | 21375.0 | 10687.5 | 5343.8 | 2671.9 | 1335.9 | 668.0 | 334.0 | 167.0 | 83.5 | 41.7 | 20.9 |
| 20-HDoHE | 21375.0 | 10687.5 | 5343.8 | 2671.9 | 1335.9 | 668.0 | 334.0 | 167.0 | 83.5 | 41.7 | 20.9 |
| 16-HDoHE | 21375.0 | 10687.5 | 5343.8 | 2671.9 | 1335.9 | 668.0 | 334.0 | 167.0 | 83.5 | 41.7 | 20.9 |
| 16,17-EpDPE | 30525.0 | 15262.5 | 7631.3 | 3815.6 | 1907.8 | 953.9 | 477.0 | 238.5 | 119.2 | 59.6 | 29.8 |
| 9-HETE | 21000.0 | 10500.0 | 5250.0 | 2625.0 | 1312.5 | 656.3 | 328.1 | 164.1 | 82.0 | 41.0 | 20.5 |
| 11,12-EpETrE | 21000.0 | 10500.0 | 5250.0 | 2625.0 | 1312.5 | 656.3 | 328.1 | 164.1 | 82.0 | 41.0 | 20.5 |
| 14-HDoHE | 21375.0 | 10687.5 | 5343.8 | 2671.9 | 1335.9 | 668.0 | 334.0 | 167.0 | 83.5 | 41.7 | 20.9 |
| 10-HDoHE | 21375.0 | 10687.5 | 5343.8 | 2671.9 | 1335.9 | 668.0 | 334.0 | 167.0 | 83.5 | 41.7 | 20.9 |
| 12-HETE | 21000.0 | 10500.0 | 5250.0 | 2625.0 | 1312.5 | 656.3 | 328.1 | 164.1 | 82.0 | 41.0 | 20.5 |
| 11-HDoHE | 21375.0 | 10687.5 | 5343.8 | 2671.9 | 1335.9 | 668.0 | 334.0 | 167.0 | 83.5 | 41.7 | 20.9 |
| 8-HDoHE | 21375.0 | 10687.5 | 5343.8 | 2671.9 | 1335.9 | 668.0 | 334.0 | 167.0 | 83.5 | 41.7 | 20.9 |
| 8-HETrE | 22800.0 | 11400.0 | 5700.0 | 2850.0 | 1425.0 | 712.5 | 356.3 | 178.1 | 89.1 | 44.5 | 22.3 |
| 5-HETrE | 22800.0 | 11400.0 | 5700.0 | 2850.0 | 1425.0 | 712.5 | 356.3 | 178.1 | 89.1 | 44.5 | 22.3 |
| 5S,14R-lipoxinB4 | 20850.0 | 10425.0 | 5212.5 | 2606.3 | 1303.1 | 651.6 | 325.8 | 162.9 | 81.4 | 40.7 | 20.4 |

**Supplementary Table 3.** Mass spectrometer parameters in the developed micro-LC-MS/MS method

| **Compound Name** | **Q1 mass**  **(*m/z*)** | **Q3 mass**  **(*m/z*)** | **Dwell time**  **(ms)** | **EP(V)** | **CE(V)** | **CXP(V)** | **Q0D(V)** | **Retention time (min)** |
| --- | --- | --- | --- | --- | --- | --- | --- | --- |
| 20-hydroxy-PGF2α | 369.2 | 325.2 | 200.00 | -12 | -28 | -15 | -25 | 10.02 |
| 20-hydroxy-PGE2 | 367.2 | 287.2 | 200.00 | -11 | -22 | -14 | -25 | 10.16 |
| 2,3dinor-8iso-PGF2α | 325.1 | 237.2 | 200.00 | -9 | -16 | -15 | -25 | 13.67 |
| 2,3dinor-11β-PGF2α | 325.0 | 145.2 | 200.00 | -9 | -23 | -13 | -25 | 14.77 |
| 20-carboxy-LTB4 | 365.2 | 347.2 | 200.00 | -10 | -23 | -16 | -25 | 16.35 |
| 20-hydroxy-LTB4 | 351.2 | 195.2 | 200.00 | -7 | -23 | -10 | -25 | 17.17 |
| iPF2α-IV | 353.3 | 127.1 | 200.00 | -11 | -29 | -14 | -25 | 18.55 |
| d_4_-8iso-PGF2α-ISTD | 357.3 | 197.15 | 200.00 | -10 | -33 | -13 | -25 | 19.46 |
| PGE3; PGD3 | 349.2 | 269.2 | 200.00 | -10 | -21 | -12 | -25 | 20.00 |
| d_11_-5-iPF2α-VI-ISTD | 364.2 | 115.05 | 169.25 | -10 | -30 | -13 | -25 | 20.45 |
| 5-iPFα-VI | 353.2 | 115.05 | 162.12 | -10 | -28 | -10 | -25 | 20.80 |
| 8iso-15R-PGF2α;8iso-PGF2α;11β-PGF2α;PGF2α | 353.1 | 193.1 | 200.00 | -10 | -33 | -10 | -25 | 21.00 |
| 9,12,13-TriHOME | 329.2 | 211.1 | 170.46 | -9 | -29 | -10 | -50 | 22.03 |
| 9,10,13-TriHOME | 329.2 | 171.1 | 127.11 | -10 | -33 | -11 | -50 | 22.43 |
| d_4_-PGF2α-ISTD | 357.3 | 197.15 | 125.20 | -10 | -33 | -13 | -25 | 22.50 |
| 8iso-13,14dihydro-15keto-PGF2α | 353.3 | 183.1 | 135.84 | -8 | -32 | -9 | -25 | 22.88 |
| d_9_-PGE2-ISTD | 360.3 | 280.25 | 152.01 | -10 | -21 | -13 | -25 | 23.13 |
| d_4_-8iso-PGE2-ISTD;d_4_-PGE2-ISTD;d_4_-PGD2-ISTD | 355.3 | 275.25 | 183.42 | -10 | -21 | -13 | -25 | 23.20 |
| 8iso-PGE2; PGE2; 11β-PGE2; PGD2 | 351.1 | 271.15 | 165.78 | -8 | -23 | -14 | -25 | 23.50 |
| 5S,14R-lipoxinB4 | 351.2 | 221.2 | 200.00 | -14 | -20 | -10 | -35 | 24.05 |
| 13,14dihydro-PGF2α | 355.2 | 311.3 | 189.05 | -13 | -31 | -17 | -25 | 24.51 |
| d_11_-8,12-iso-iPF2α-VI-ISTD | 364.21 | 115.05 | 148.55 | -10 | -30 | -13 | -25 | 25.10 |
| 8,12-iso-iPF2α-VI | 353.21 | 115.05 | 144.80 | -7 | -25 | -13 | -25 | 25.20 |
| 5S,6S-LipoxinA4 | 351.21 | 115.2 | 153.12 | -13 | -20 | -13 | -35 | 25.41 |
| 13,14dihydro-15keto-PGE2 | 351.2 | 175.1 | 160.38 | -13 | -30 | -8 | -25 | 25.52 |
| 13,14dihydro-15keto-PGF2α | 353.31 | 183.1 | 161.00 | -11 | -35 | -16 | -25 | 25.53 |
| 5S,6R-LipoxinA4 | 351.2 | 115.2 | 200.00 | -14 | -35 | -10 | -35 | 25.91 |
| 1a-1b-dihomo-PGF2α | 381.1 | 337.45 | 200.00 | -13 | -29 | -13 | -25 | 26.43 |
| 13,14dihydro-15keto-PGD2 | 351.21 | 175.1 | 200.00 | -11 | -27 | -13 | -25 | 26.59 |
| 8,15-DiHETE | 335.2 | 235.1 | 200.00 | -13 | -12 | -11 | -50 | 28.47 |
| bicyclo-PGE2 | 333.2 | 113.15 | 200.00 | -13 | -33 | -13 | -25 | 28.75 |
| 17,18-DiHETE | 335.2 | 247.1 | 185.52 | -8 | -24 | -11 | -50 | 28.98 |
| 10,17-DiHDoHE | 359.2 | 153.1 | 176.57 | -13 | -24 | -14 | -50 | 29.06 |
| d_4_-LTB4-ISTD | 339.5 | 197.1 | 171.63 | -10 | -20 | -13 | -50 | 29.18 |
| 14,15-DiHETE | 335.2 | 207.1 | 161.35 | -7 | -22 | -10 | -50 | 29.57 |
| d_4_-12,13-DiHOME-ISTD | 317.2 | 185.1 | 142.71 | -10 | -30 | -13 | -50 | 29.95 |
| 12,13-DiHOME | 313.2 | 183.1 | 144.99 | -11 | -30 | -11 | -50 | 30.04 |
| d_4_-9,10-DiHOME-ISTD | 317.2 | 203.1 | 151.84 | -10 | -30 | -13 | -50 | 30.38 |
| d_11_-14,15-DiHETrE-ISTD | 348.2 | 207.1 | 176.19 | -10 | -24 | -13 | -50 | 31.20 |
| 14,15-DiHETrE | 337.2 | 207.1 | 178.48 | -9 | -24 | -9 | -50 | 31.30 |
| 12-HHTrE | 279.2 | 179.1 | 189.13 | -11 | -18 | -10 | -50 | 31.40 |
| 19,20-DiHDPa | 361.2 | 273.2 | 200.00 | -10 | -21 | -13 | -50 | 31.50 |
| 11,12-DiHETrE | 337.2 | 167.3 | 200.00 | -7 | -24 | -14 | -50 | 32.20 |
| 9-HOTrE | 293.2 | 171.2 | 162.00 | -9 | -22 | -10 | -50 | 32.64 |
| 8,9-DiHETrE | 337.2 | 127.2 | 152.47 | -10 | -27 | -14 | -50 | 32.67 |
| 18-HEPE | 317.2 | 299.2 | 98.84 | -11 | -18 | -14 | -50 | 32.92 |
| 5,6-DiHETrE | 337.2 | 145.15 | 59.84 | -8 | -24 | -13 | -50 | 33.16 |
| d_6_-20-HETE-ISTD | 325.2 | 279.2 | 55.36 | -10 | -21 | -13 | -50 | 33.24 |
| 15-HEPE | 317.2 | 219.2 | 54.70 | -8 | -18 | -11 | -50 | 33.26 |
| 20-HETE | 319.2 | 289.2 | 53.90 | -8 | -24 | -13 | -50 | 33.28 |
| 12-HEPE | 317.2 | 179.1 | 48.05 | -9 | -18 | -14 | -50 | 33.45 |
| 9-HEPE | 317.2 | 167.25 | 46.42 | -13 | -18 | -14 | -50 | 33.51 |
| 5-HEPE | 317.2 | 115.2 | 43.36 | -8 | -12 | -13 | -50 | 33.64 |
| d_4_-9-HODE-ISTD | 299.2 | 172.1 | 40.81 | -10 | -27 | -13 | -50 | 33.74 |
| 9-HODE | 295.21 | 171.1 | 40.81 | -8 | -15 | -15 | -50 | 33.74 |
| 13-HODE | 295.2 | 195.2 | 40.81 | -10 | -15 | -14 | -50 | 33.75 |
| 20-HDoHE | 343.21 | 299.2 | 40.25 | -15 | -18 | -16 | -50 | 33.85 |
| 16-HDoHE | 343.2 | 233.2 | 39.63 | -12 | -18 | -10 | -45 | 34.02 |
| 16,17-EpDPE | 343.21 | 233.2 | 39.63 | -10 | -15 | -11 | -50 | 34.02 |
| 11,12-EpETrE | 319.22 | 167.1 | 40.61 | -10 | -18 | -15 | -45 | 34.12 |
| 9-HETE | 319.21 | 167.1 | 40.91 | -9 | -10 | -15 | -50 | 34.13 |
| 10-HDoHE | 343.21 | 153.1 | 42.16 | -14 | -21 | -15 | -40 | 34.17 |
| 14-HDoHE | 343.2 | 205.1 | 42.16 | -7 | -18 | -15 | -50 | 34.18 |
| d_8_-12-HETE-ISTD | 327.2 | 184.1 | 43.76 | -10 | -21 | -13 | -50 | 34.21 |
| 12-HETE | 319.2 | 179.2 | 47.17 | -12 | -10 | -14 | -50 | 34.28 |
| 11-HDoHE | 343.2 | 121.1 | 47.17 | -11 | -18 | -14 | -45 | 34.28 |
| 8-HDoHE | 343.2 | 189.1 | 55.57 | -14 | -15 | -15 | -45 | 34.40 |
| 8-HETrE | 321.2 | 303.2 | 55.57 | -12 | -18 | -16 | -40 | 34.40 |
| 17-HDoHE | 343.2 | 281.2 | 103.43 | -13 | -16 | -13 | -50 | 34.50 |
| 5-HETrE | 321.21 | 303.2 | 79.86 | -7 | -18 | -15 | -50 | 34.55 |
| 12,13-EpOME | 295.21 | 195.2 | 167.46 | -7 | -21 | -13 | -45 | 34.90 |
| 9,10-EpOME | 295.22 | 171.1 | 200.00 | -8 | -21 | -9 | -50 | 34.98 |

**Supplementary Table 4.** UHPLC gradients used in the comparison experiment

Mobile phase A: H_2_O with 0.1 % acetic acid, mobile phase B: 90 % ACN/10 % MeOH with 0.1 % acetic acid, mobile phase C: IPA with 0.1 % acetic acid with pH ranges between 3.2 and 3.5

| **Time [min]** | **Flow [mL/min]** | **B.Conc [%]** | **C.Conc [%]** |
| --- | --- | --- | --- |
| 0.75 | 0.70 | 20.0 | 1.0 |
| 0.95 | 0.70 | 26.0 | 1.0 |
| 6.00 | 0.70 | 34.0 | 1.0 |
| 8.00 | 0.70 | 40.0 | 1.0 |
| 10.00 | 0.70 | 54.0 | 1.0 |
| 11.00 | 0.70 | 55.0 | 1.0 |
| 12.00 | 0.70 | 56.0 | 3.0 |
| 13.00 | 0.70 | 78.0 | 6.0 |
| 14.00 | 0.70 | 85.0 | 15.0 |
| 14.50 | 0.70 | 85.0 | 15.0 |
| 14.80 | 0.70 | 20.0 | 1.0 |
| 16.00 | 0.70 | 20.0 | 1.0 |

**Supplementary Table 5.** Comparison of LODs and LOQs of micro flow-LC-MS/MS and UHPLC-MS/MS methods

| **Compounds** | **LOD (pM)** | | **Fold Change** | **LOQ (pM)** | |
| --- | --- | --- | --- | --- | --- |
|  | micro LC-MS/MS | UHPLC-MS/MS |  | micro LC-MS/MS | UHPLC-MS/MS |
| 13-HODE | 59.0 | - | - | 196.6 | - |
| 8,12-iPF2α-IV | 23.0 | - | - | 76.5 | - |
| 13,14dihydro-15keto-PGE2 | 0.3 | - | - | 0.9 | - |
| 19,20-DiHDPA | 0.1 | 19.9 | 180.7 | 0.4 | 66.3 |
| 5S,6S-LipoxinA4 | 0.1 | 16.9 | 170.2 | 0.3 | 56.2 |
| iPF2α-IV | 0.2 | 28.0 | 114.4 | 0.8 | 93.2 |
| 2,3dinor-8iso-PGF2α | 0.3 | 34.6 | 102.3 | 1.1 | 115.4 |
| PGE3 | 0.1 | 7.4 | 71.4 | 0.3 | 24.7 |
| 8iso-PGE2 | 0.1 | 4.9 | 56.9 | 0.3 | 16.3 |
| 11,12-DiHETrE | 0.1 | 5.6 | 50.8 | 0.4 | 18.6 |
| PGF2α | 0.2 | 11.6 | 48.4 | 0.8 | 38.7 |
| 15-HEPE | 0.4 | 13.7 | 31.2 | 1.5 | 45.8 |
| 2,3dinor-11β-PGF2α | 1.2 | 29.4 | 23.8 | 4.1 | 98.1 |
| 8iso-13,14dihydro-15keto-PGF2α | 1.1 | 24.7 | 23.0 | 3.6 | 82.5 |
| 11β-PGE2 | 0.3 | 6.1 | 21.4 | 0.9 | 20.2 |
| 13,14dihydro-15keto-PGD2 | 0.3 | 6.7 | 21.1 | 1.1 | 22.2 |
| 20-hydroxy-LTB4 | 0.8 | 16.1 | 20.8 | 2.6 | 53.8 |
| 20-carboxy-LTB4 | 1.3 | 23.5 | 18.6 | 4.2 | 78.4 |
| 8,9-DiHETrE | 0.5 | 8.5 | 18.0 | 1.6 | 28.2 |
| 5S,6R-lipoxinA4 | 1.0 | 18.1 | 18.0 | 3.4 | 60.4 |
| 17,18-DiHETE | 2.4 | 42.7 | 17.7 | 8.0 | 142.4 |
| 8iso-PGF2α | 2.1 | 31.3 | 15.3 | 6.8 | 104.3 |
| 12-HEPE | 0.4 | 6.1 | 13.9 | 1.5 | 20.3 |
| 9,10-EpOME | 2.2 | 28.5 | 13.0 | 7.3 | 94.9 |
| 9,12,13-TriHOME | 6.6 | 69.1 | 10.5 | 22.0 | 230.5 |
| 14,15-DiHETE | 0.7 | 6.8 | 10.0 | 2.3 | 22.5 |
| 16,17-EpDPE | 5.1 | 50.1 | 9.9 | 16.8 | 167.1 |
| 20-hydroxy-PGE2 | 0.3 | 2.5 | 8.9 | 1.0 | 8.5 |
| 18-HEPE | 3.0 | 18.7 | 6.3 | 9.9 | 62.4 |
| 12,13-DiHOME | 2.3 | 14.2 | 6.2 | 7.6 | 47.2 |
| 5,6-DiHETrE | 2.2 | 13.3 | 6.0 | 7.4 | 44.3 |
| 11β-PGF2α | 1.1 | 6.6 | 5.8 | 3.8 | 22.0 |
| PGE2 | 0.9 | 4.9 | 5.6 | 2.9 | 16.2 |
| 8-HETrE | 4.4 | 21.2 | 4.9 | 14.5 | 70.5 |
| PGD2 | 0.4 | 1.8 | 4.8 | 1.3 | 6.1 |
| PGD3 | 0.6 | 2.5 | 4.6 | 1.8 | 8.4 |
| 1a,1b-dihomo-PGF2α | 0.6 | 2.5 | 4.0 | 2.1 | 8.4 |
| 10,17-DiHDoHE | 4.7 | 17.4 | 3.7 | 15.8 | 57.9 |
| 8iso-15R-PGF2α | 0.8 | 3.0 | 3.6 | 2.8 | 9.9 |
| 9-HEPE | 3.9 | 13.5 | 3.5 | 12.9 | 45.0 |
| 9-HOTrE | 2.6 | 9.1 | 3.4 | 8.8 | 30.4 |
| 14,15-DiHETrE | 0.9 | 2.9 | 3.3 | 3.0 | 9.8 |
| 8,15-DiHETE | 6.1 | 17.9 | 2.9 | 20.5 | 59.8 |
| bicyclo-PGE2 | 0.9 | 2.6 | 2.9 | 3.0 | 8.8 |
| 13,14dihydro-PGF2α | 3.5 | 9.7 | 2.8 | 11.7 | 32.4 |
| 12-HHTrE | 4.4 | 12.2 | 2.8 | 14.7 | 40.7 |
| 11,12-EpETrE | 0.6 | 1.5 | 2.7 | 1.8 | 5.0 |
| 20-hydroxy-PGF2α | 1.9 | 5.0 | 2.7 | 6.2 | 16.6 |
| 5-iPF2α-VI | 2.5 | 6.6 | 2.6 | 8.3 | 22.0 |
| 12,13-EpOME | 4.3 | 10.6 | 2.5 | 14.4 | 35.3 |
| 5-HETrE | 4.7 | 10.4 | 2.2 | 15.6 | 34.7 |
| 9,10,13-TriHOME | 4.6 | 9.5 | 2.1 | 15.4 | 31.8 |
| 14-HDoHE | 7.1 | 10.4 | 1.5 | 23.6 | 34.5 |
| 20-HETE | 8.8 | 12.5 | 1.4 | 29.3 | 41.8 |
| 13,14dihydro-15keto-PGF2α | 2.5 | 2.4 | 1.0 | 8.5 | 8.1 |
| 5S,14R-lipoxinB4 | 11.6 | 11.0 | 0.9 | 38.6 | 36.7 |
| 9-HETE | 8.3 | 7.0 | 0.8 | 27.8 | 23.3 |
| 11-HDoHE | 8.8 | 6.8 | 0.8 | 29.2 | 22.8 |
| 10-HDoHE | 12.8 | 9.4 | 0.7 | 42.6 | 31.3 |
| 9-HODE | 27.7 | 17.8 | 0.6 | 92.3 | 59.3 |
| 16-HDoHE | 3.4 | 1.9 | 0.5 | 11.5 | 6.2 |
| 5-HEPE | 5.7 | 2.3 | 0.4 | 19.1 | 7.7 |
| 12-HETE | 21.9 | 6.3 | 0.3 | 72.9 | 21.0 |
| 17-HDoHE | 47.3 | 6.4 | 0.1 | 157.6 | 21.5 |
| 20-HDoHE | 91.9 | 10.3 | 0.1 | 306.2 | 34.5 |
| 8-HDoHE | 76.4 | 4.7 | 0.1 | 254.6 | 15.8 |


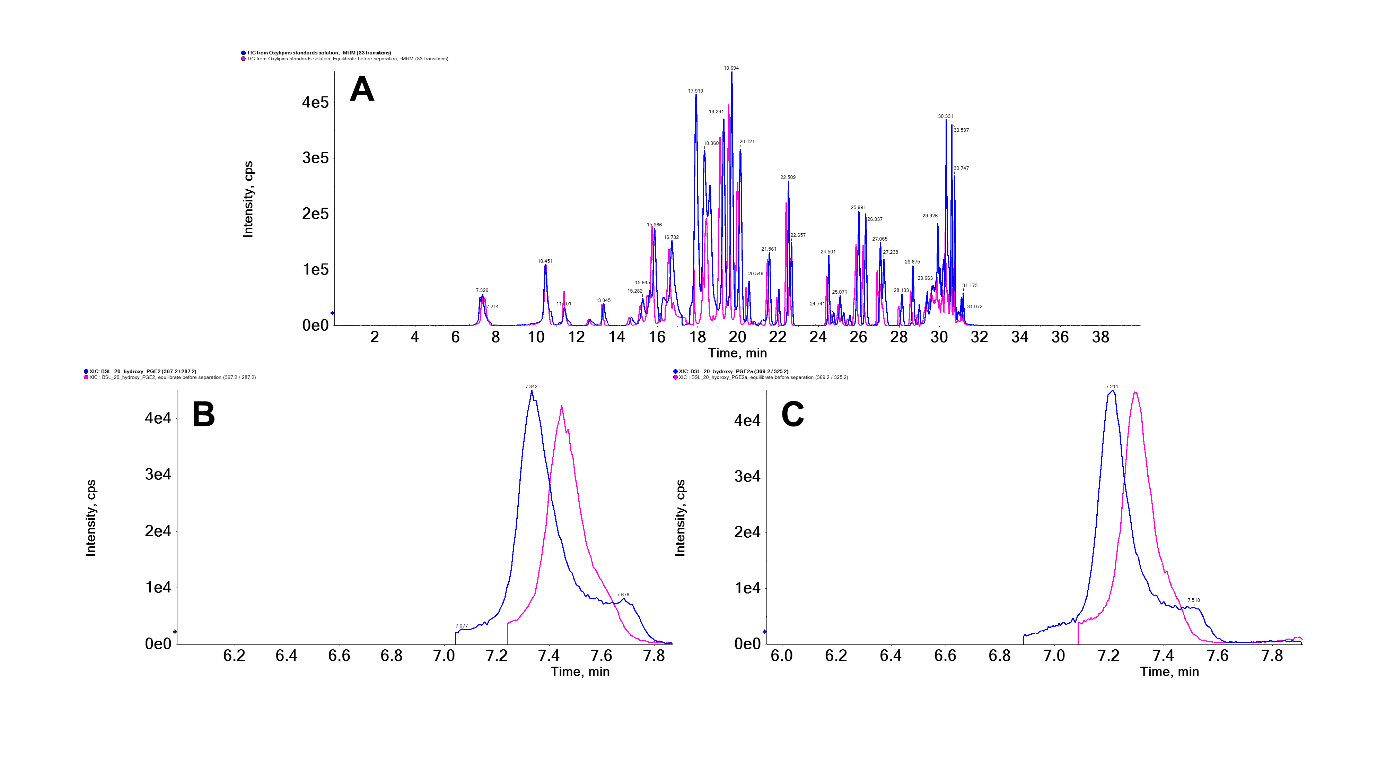
**Supplementary Figure 1.** Effect of equilibration time on early eluted compounds. A. Total ion chromatogram from injections of oxylipins standard solution; B. Extracted SRM chromatograms of 20-hydroxy-PGE2; C. Extracted SRM chromatograms of 20-hydroxy-PGF2α. Blue line represents the analysis with 1.5-min initial gradient, red line represents analysis with 5.5-min initial gradient.
